# Designed synthesis of stable light-emitting two-dimensional sp^2^ carbon-conjugated covalent organic frameworks

**DOI:** 10.1038/s41467-018-06719-8

**Published:** 2018-10-08

**Authors:** Enquan Jin, Juan Li, Keyu Geng, Qiuhong Jiang, Hong Xu, Qing Xu, Donglin Jiang

**Affiliations:** Department of Chemistry, Faculty of Science, National University of Singapore, 3 Science Drive 3, Singapore, 117543 Singapore

## Abstract

Covalent organic frameworks enable the topological connection of organic chromophores into π lattices, making them attractive for creating light-emitting polymers that are predesignable for both the primary- and high-order structures. However, owing to linkages, covalent organic frameworks are either unstable or poor luminescent, leaving the practical synthesis of stable light-emitting frameworks challenging. Here, we report the designed synthesis of sp^2^ carbon-conjugated frameworks that combine stability with light-emitting activity. The C=C linkages topologically connect pyrene knots and arylyenevinylene linkers into two-dimensional all sp^2^ carbon lattices that are designed to be π conjugated along both the *x* and *y* directions and develop layer structures, creating exceptionally stable frameworks. The resulting frameworks are capable of tuning band gap and emission by the linkers, are highly luminescent under various conditions and can be exfoliated to produce brilliant nanosheets. These results suggest a platform based on sp^2^ carbon frameworks for designing robust photofunctional materials.

## Abstract (web summary)

Topological connection of organic chromophores is an attractive way to design light-emitting covalent organic frameworks but the synthesis of stable light-emitting frameworks remains challenging. Here the authors report the designed synthesis of sp2 carbon conjugated frameworks that combine stability with light-emitting activity

## Introduction

Covalent organic frameworks (COFs) are a class of crystalline porous polymer with predesignable lattice structures of organic building blocks^1–8^. COFs can be predesigned by using topological diagram that enables the development of various lattices based on the combination of building units with different geometries^9–25^. A diversity of chromophores has been explored for the synthesis of photofunctional and semiconducting COFs of different space groups^26–40^. However, owing to the shortage of a suitable linkage that combines stability with luminescence, stable COFs are less luminescent and luminescent COFs are chemically unstable. For example, boronate and boroxine linkages can form luminescent COFs but they are unstable under protic conditions or upon exposure to air^26–30^, while imine linkages are most widely used for the preparation of relatively stable frameworks that however, are less or not luminescent^14,31,32^. Developing a linkage to design COFs that combine luminescence activity and stability remains challenging.

In this work, we developed a C=C linkage for the construction of two-dimensional (2D) sp^2^ carbon-conjugated frameworks (sp^2^c-COFs) that are designed to be π conjugated along both the *x* and *y* directions and develop eclipsed layer structures. The C=C polycondensations of tetraphenylpyrene knots and phenyl, biphenyl, and terphenyl linkers enable the designed synthesis of sp^2^c-COFs with different π lattice sizes. The resulting sp^2^ carbon frameworks are exceptionally stable under various conditions upon immersion in acid and base solutions or exposure to air over a long period. We observed that the linkers adopt propeller conformation to constitute twisted arylenevinylene backbones so that the transmission of π delocalization along the *x* and *y* directions can be controlled by the linker units, enabling the tune of both band gap and emission colors. We elucidated the structure-photofunction correlation by disclosing the structural parameters that control photochemical events. We highlighted that the resulting frameworks are highly luminescent under various conditions and can be exfoliated to produce light-emitting sp^2^ carbon nanosheets.

## Results

### Molecular design

Among various topologies, we explored a tetragonal topology for designing the sp^2^ carbon frameworks since it enables π conjugation along both the *x* and *y* directions to transmit the π cloud overlap over the 2D skeleton^41^. Among various linkages for the synthesis of COFs, recently we have developed a C=C linkage for synthesizing sp^2^ carbon-conjugated frameworks^42^. The C=C linkage is different from the boronate, boroxine, imine, hydrozone, imide, and phenazine bonds since it allows for the formation of fully conjugated systems from all sp^2^ carbon. In this study, we successfully explored the C=C linkage for the designed synthesis of sp^2^ carbon-conjugated COFs and disclosed their stability and photofunctionality.

### Synthesis

**Fig. 1:**
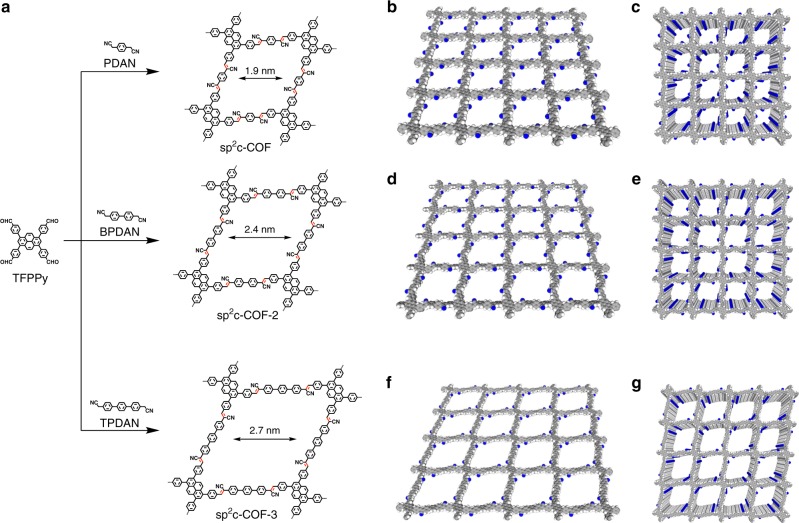
Design and structures. **a** Syntheses of sp^2^c-COF through condensation of TFPPy and PDAN, sp^2^c-COF-2 through condensation of TFPPy and BPDAN and sp^2^c-COF-3 through condensation of TFPPy and TPDAN. **b** Reconstructed crystal structure of single layer of sp^2^c-COF. **c** Reconstructed crystal structure of many layers of sp^2^c-COF. **d** Reconstructed crystal structure of single layer of sp^2^c-COF-2. **e** Reconstructed crystal structure of many layers of sp^2^c-COF-2. **f** Reconstructed crystal structure of single layer of sp^2^c-COF-3. **g** Reconstructed crystal structure of many layers of sp^2^c-COF-3

We selected the *C*_2_+*C*_2_ topology diagram for the construction of 2D tetragonal sp^2^ carbon frameworks. The C=C polycondensation of 1,3,6,8-tetrakis(4-formylphenyl)pyrene (TFPPy) as knots with 2,2’-(1,4-phenylene)diacetonitrile (PDAN), 2,2’-(biphenyl-4,4’-diyl)diacetonitrile (BPDAN), and 2,2’-([1,1’:4’,1”-terphenyl]-4,4”-diyl)diacetonitrile (TPDAN) as linkers topologically connected pyrene units at regular intervals of 1.9, 2.4, and 2.8 nm into sp^2^ carbon lattices and develop eclipsed layer frameworks of sp^2^c-COF, sp^2^c-COF-2, and sp^2^c-COF-3, respectively (Fig. 1). To achieve high crystallinity, we optimized the reaction conditions, including solvents, reaction temperatures, and catalysts (ESI). High quality sp^2^c-COF and sp^2^c-COF-2 crystallites were prepared in 84% and 82% isolated yields by condensation in 1,4-dioxane in the presence of KOH catalyst at 90 °C and 110 °C, respectively. The polycondensation of TFPPy and TPDAN in *o*-dichlorobenzene (*o*-DCB) in the presence of a methanol (MeOH) solution of tetrabutylammonium hydroxide (TBAH) as catalyst for 3 days at 120 °C produced sp^2^c-COF-3 in 88% isolated yield. The chemical structures of sp^2^c-COFs were unambiguously characterized by various analytical methods (Supplementary Figures 1–21). Fourier transform infrared spectroscopy (FT IR) revealed the formation of sp^2^ carbon-conjugated COFs by showing a newly appeared C≡N vibration band at 2220 cm^–1^ and a greatly decreased vibration band at 2720 cm^–1^ that was assigned to the C–H stretching vibration of the aldehyde units (Suppelementary Fig. 1). Field-emission scanning electron microscope revealed that these sp^2^ carbon COFs were similar in morphology to assume belt shapes (Supplementary Figure 2). High resolution transmission electron microscope (HR-TEM) image revealed the tetragonal and layer structure (Supplementary Figure 3). The FFT and line profiles further indicate the lattice structures that clearly show a pore size of 2 nm and an interlayer separation of 3.5 Å.

### Stability of COFs

**Fig. 2:**
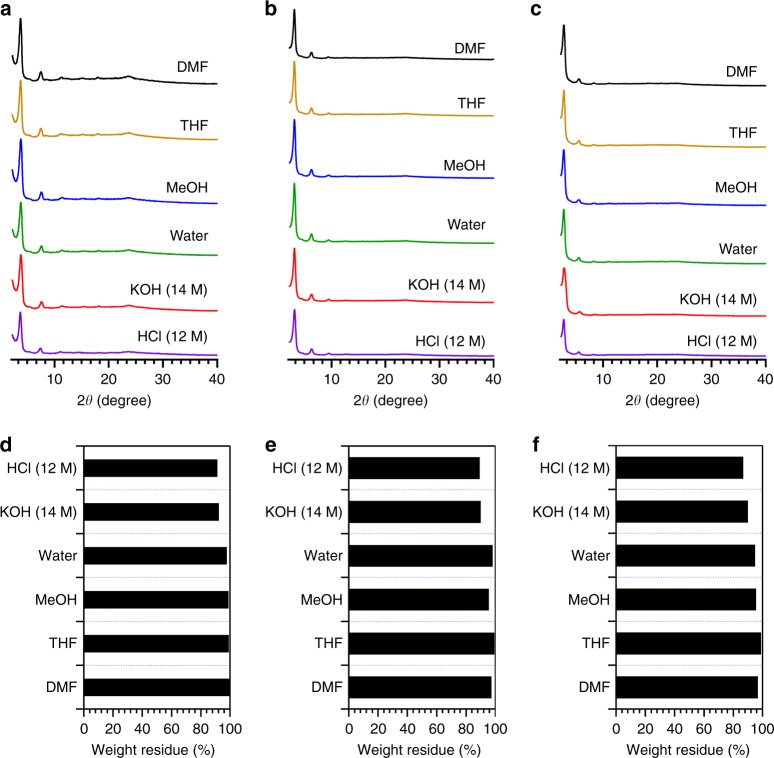
Stability. **a**–**c** PXRD patterns of **a** sp^2^c-COF, **b** sp^2^c-COF-2, and **c** sp^2^c-COF-3 after treatment in different solvents for 1 week. **d**–**f** Residual weight percentage of **d** sp^2^c-COF, **e** sp^2^c-COF-2, and **f** sp^2^c-COF-3 after treatment in different solvents for 1 week

To evaluate their chemical stability, we immersed sp^2^c-COF, sp^2^c-COF-2, and sp^2^c-COF-3 for 1 week in various solvents, including tetrahydrofuran (THF), *N*,*N*’-dimethylformamide (DMF), MeOH, water, concentrated HCl (12 M) and aqueous NaOH (14 M) solutions (ESI). Notably, these sp^2^ carbon COFs retained their original powder X-ray diffraction (PXRD) profiles (Fig. 2a–c). From the residual weight percentages, they exhibited nearly no weight loss (<0.1 wt%) in water and organic solvents (Fig. 2d–f). Even under strong acid (12 M HCl) and base (14 M NaOH) conditions, the residual weight percentages were as high as 90 wt%. FT IR spectroscopy revealed that the chemical structures were preserved (Supplementary Figure 4).

**Fig. 3:**
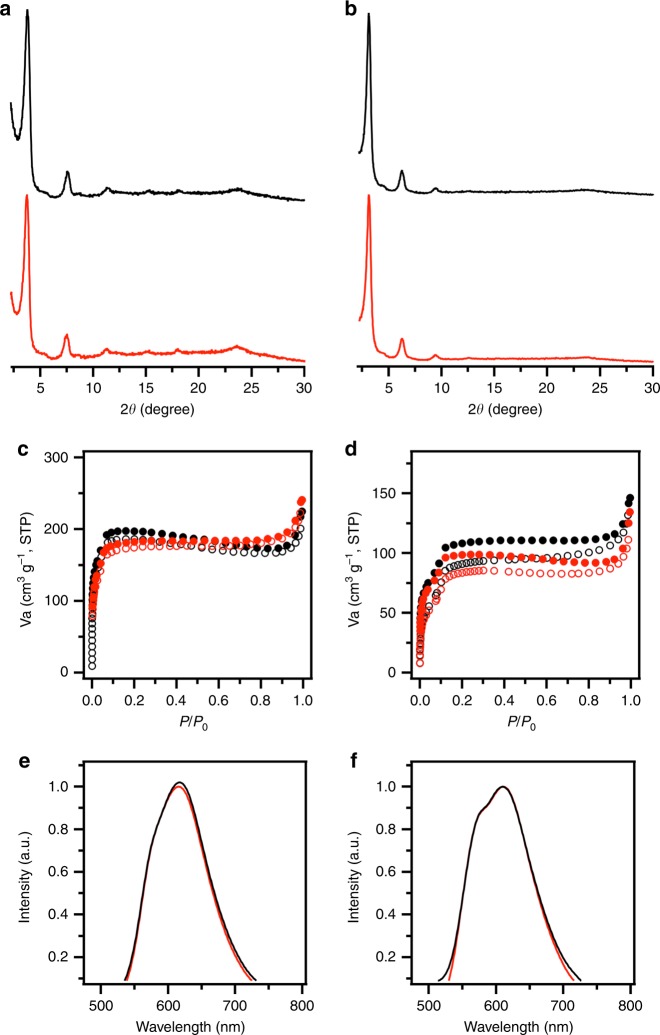
Stability in air. **a**, **b** PXRD patterns of **a** sp^2^c-COF and **b** sp^2^c-COF-2 upon exposure to air over 1 year. **c**, **d** Nitrogen sorption isotherm curves of **c** sp^2^c-COF and **d** sp^2^c-COF-2 upon 1-year exposure to air. **e**, **f** Fluorescence spectra of **e** sp^2^c-COF and **f** sp^2^c-COF-2 upon exposure to air over 1 year. Black and red curves are samples before and after exposure to air

Surprisingly, we observed that the sp^2^ carbon frameworks are stable to keep crystallinity and porosity upon long-term exposure to air at room temperature. For example, both sp^2^c-COF and sp^2^c-COF-2 upon exposure to air for 1 year retained their structures by showing nearly the same intensities and peak positions in the PXRD patterns (Fig. 3a, b). The sp^2^c-COF and sp^2^c-COF-2 samples can keep their porosity; after 1-year exposure to air they exhibited the Brunauer–Emmett–Teller (BET) surface areas as high as 581 and 302 m^2^ g^–1^, respectively (Fig. 3c, d, red curves), which are close to those (sp^2^c-COF; 613 m^2^ g^–1^, sp^2^c-COF-2; 322 m^2^ g^–1^) of the as-synthesized samples (Fig. 3c, d, black curves). Pore size distribution profiles revealed that the pore size did not change (Supplementary Figure 5a–d). FT IR spectra revealed that the chemical structures were retained (Supplementary Figure 6a–d).

Along this line, we further investigated luminescent properties of sp^2^c-COF and sp^2^c-COF-2 upon 1-year exposure to air. Surprisingly, the sample retained its luminescent wavelength (Fig. 3e, f), absolute fluorescence quantum yield (sp^2^c-COF, 14% for as-synthesized versus 12% after 1-year exposure to air; sp^2^c-COF-2, 10% for as-synthesized versus 9% after 1-year exposure to air) and lifetime (Supplementary Figures 7 and 8). To the best of our knowledge, the sp^2^ carbon-conjugated COFs are the most stable and luminescent ones among all the COFs reported to date^26–40^. As a control, we investigated an imine-linked pyrene COF with a similar structure to that of sp^2^c-COF (Supplementary Figure 9). However, the imine-linked pyrene COF upon 1-year exposure to air cannot retain its crystallinity; in this case nearly no clear PXRD pattern was observable.

### Crystal structure

**Fig. 4:**
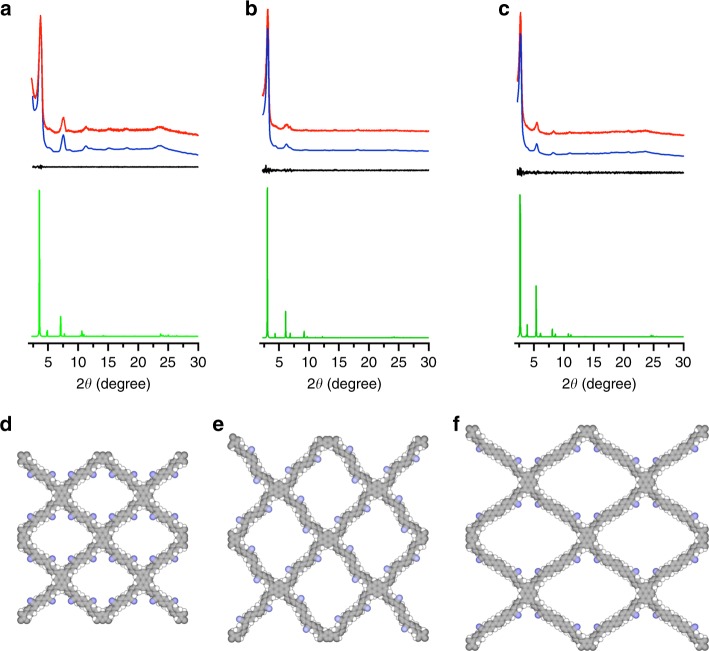
Crystal structure. **a**–**c** PXRD profiles of **a** sp^2^c-COF, **b** sp^2^c-COF-2, and **c** sp^2^c-COF-3. Experimentally observed (red curve), Pawley refined (blue curve), their difference (black curve), and simulated AA stacking mode (green curve). **d**–**f** Lattice structure of **d** sp^2^c-COF, **e** sp^2^c-COF-2, and **f** sp^2^c-COF-3

The PXRD patterns revealed that sp^2^c-COF, sp^2^c-COF-2, and sp^2^c-COF-3 were highly crystalline polymers (Fig. 4a–c, red curves). The sp^2^c-COF exhibited the PXRD peaks at 3.6°, 5.2°, 5.9°, 7.3°, 11.2°, 14.2°, and 24.7°, which were assigned to the (110), (200), (210), (220), (240), (520), and (001) facets, respectively (Fig. 4a, red curve). For sp^2^c-COF-2, the PXRD peaks at 3.1°, 5.9°, 6.8°, and 23.8° were assignable to the (110), (210), (120), and (001) facets, respectively (Fig. 4a, red curve). In the case of sp^2^c-COF-3, the PXRD peaks were observed at 2.7°, 5.5°, 8.2°, 10.9°, and 23.5°, which were attributed to the (110), (220), (240), (440), and (001) facets, respectively (Fig. 4c, red curve). The Pawley refined PXRD patterns of sp^2^c-COF, sp^2^c-COF-2, and sp^2^c-COF-3 (Fig. 4a–c, blue curves) can reproduce their experimentally observed curves with a negligible difference (Fig. 4a–c, black curves). sp^2^c-COF has a *C*2/*m* space group with the cell unit parameters of *a* = 33.9309 Å, *b* = 34.8219 Å, *c* = 3.7676 Å, *α* = 90°, *β* = 106.6756°, and *γ* = 90° (Supplementary Tables 1 and 2). On the other hand, sp^2^c-COF-2 adopts a *P*1 space group with the cell unit parameters of *a* = 29.1468 Å, *b* = 28.6019 Å, *c* = 3.7293 Å, *α* = 89.7834°, *β* = 90.2665°, and *γ* = 89.0697 (Supplementary Tables 3 and 4), while sp^2^c-COF-3 possesses a *C*2/*m* space group with the cell unit parameters of *a* = 48.8867 Å, *b* = 45.8095 Å, *c* = 3.6802 Å, *α* = 90°, *β* = 103.0632°, and *γ* = 90° (Supplementary Tables 5 and 6).

Among various possible stacking modes, the AA stacking mode is the most stable form in energy. The AA stacking mode yielded the PXRD patterns (Fig. 4a–c, green curves) that were in an agreement with the experimentally observed profiles. The AB stacking models cannot reproduce the experimentally observed PXRD patterns (Supplementary Figure 10, purple curves). Consequently, the 2D polymers adopt a tetragon skeleton with interweaved sp^2^ carbon-conjugated backbones and stack to form one type of tetragonal channels with the theoretical pore sizes of 1.9, 2.4, and 2.7 nm, respectively (Fig. 4d–f). The presence of the (001) facets suggests that the structural ordering is extended to the third dimension perpendicular to the 2D sheets. Based on the (001) facets, the interlayer distance of sp^2^c-COF, sp^2^c-COF-2, and sp^2^c-COF-3 was evaluated to be 3.58, 3.74, and 3.79 Å, respectively. The increased interlayer separation of sp^2^c-COF-2 and sp^2^c-COF-3 compared to that of sp^2^c-COF originates from the twisted biphenyl and terphenyl linkers in the frameworks.

### Porosity

The sp^2^c-COF, sp^2^c-COF-2, and sp^2^c-COF-3 samples exhibited reversible nitrogen sorption isotherm curves (Supplementary Figure 11). The BET surface areas were evaluated to be as high as 613, 322, and 737 m^2^ g^–1^ for sp^2^c-COF, sp^2^c-COF-2, and sp^2^c-COF-3, respectively. The sp^2^c-COF, sp^2^c-COF-2, and sp^2^c-COF-3 samples have pore volumes of 0.30, 0.14, and 0.32 cm^3^ g^–1^, respectively. The pore size distribution profiles calculated using nonlinear density functional theory (NLDFT) revealed that sp^2^c-COF, sp^2^c-COF-2, and sp^2^c-COF-3 have one type of pores. sp^2^c-COF is a microporous material with the pore size of 1.90 nm, while sp^2^c-COF-2 and sp^2^c-COF-3 are mesoporous polymers with the pore size of 2.38 and 2.69 nm, respectively.

### Electronic absorption and π conjugation

**Fig. 5:**
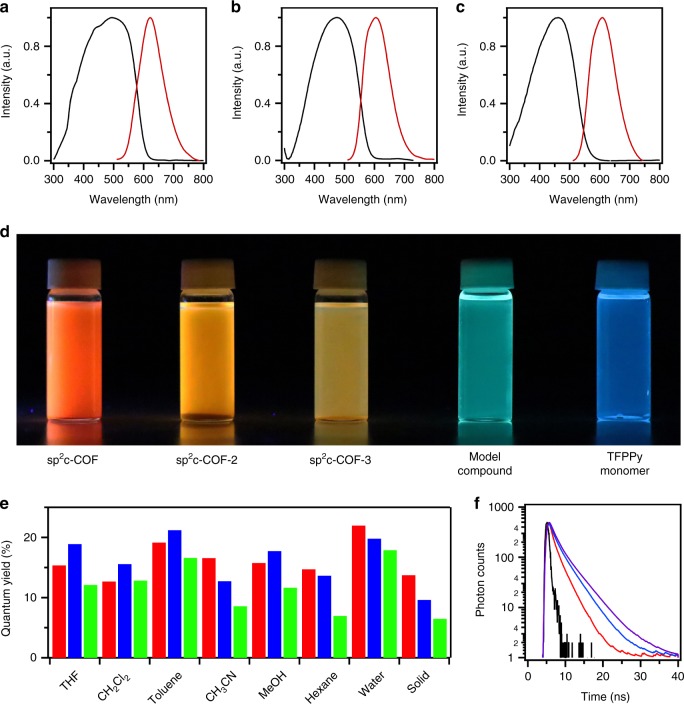
Light-emitting properties of sp^2^c-COFs. **a**–**c** Solid state absorption spectra (black curves) and fluorescence spectra (red curves) of **a** sp^2^c-COF, **b** sp^2^c-COF-2, and **c** sp^2^c-COF-3. **d** Photo images of sp^2^c-COF, sp^2^c-COF-2, sp^2^c-COF-3, model compound and TFPPy monomer dispersed in water under a UV lamp. **e** Absolute fluorescence quantum yields of sp^2^c-COF (red bars), sp^2^c-COF-2 (blue bars), and sp^2^c-COF-3 (black bars) in different solvents and in the solid state. **f** Fluorescence decay curves of sp^2^c-COF (purple curve), sp^2^c-COF-2 (blue curve), and sp^2^c-COF-3 (red curve). Black curve is the instrumental response function

The solid sample of sp^2^c-COF exhibited an electronic absorption band at 498 nm (Fig. 5a, black curve, Table 1). By contrast, the model compound exhibited an absorption band at 445 nm (Supplementary Figure 12, black curve). This 53-nm redshift in the electronic absorption band reflects extended π-conjugation over the 2D sp^2^ carbon framework. Since the imine-linked (C=N) pyrene COF has an absorption band at 477 nm^42^, the 21-nm redshift indicates that the C=C linkage is more efficient than the C=N bond in transmitting π conjugation over the 2D skeleton.

**Table 1:** Absorption and luminescence properties

	Bulk solid				Exfoliated thin film			
	Abs (nm)	Emission (nm)	QY (%)	Lifetime (ns)	Abs (nm)	Emission (nm)	QY (%)	Lifetime (ns)
sp^2^c-COF	498	622	14	1.9	437	565	14	1.9
sp^2^c-COF-2	475	606	10	2.9	418	468	21	1.7
sp^2^c-COF-3	462	609	6	3.4	438	551	16	2.0

*ABS* absorption, *QY* absolute fluorescence quantum yield

Notably, integration of the biphenyl and terphenyl units as linkers into the 2D sp^2^ carbon skeleton enables the tuning of electronic absorption bands. For example, sp^2^c-COF-2 exhibited an absorption band at 475 nm, which is 17-nm blueshifted from that of sp^2^c-COF (Fig. 5b, black curve, Table 1). More remarkably, sp^2^c-COF-3 displayed an absorption maximum at 462 nm that is 36-nm redshifted from that of sp^2^c-COF (Fig. 5c, black curve, Table 1). These blueshifts are clearly associated with the twisted structures of the linkers.

As shown in the crystal structure, sp^2^c-COF, sp^2^c-COF-2, and sp^2^c-COF-3 possess the phenyl, biphenyl and terphenyl units that connect the pyrene knots. Owing to the propeller alignment of the four phenyl units (the twisted angle is 37.81° for sp^2^c-COF, 30.45° for sp^2^c-COF-2 and 37.12° for sp^2^c-COF-3) at the focal pyrene core, the phenyl, biphenyl and terphenyl units adopt twisted confirmation relative to the pyrene plane. The phenyl linker has a twisted angle of 27.50° relative to the 2D plane, while the biphenyl unit has the twisted angles of 31.77° and 11.57° for each phenyl unit and the terphenyl linker adopts the twisted angles of 16.16°, 7.77°, and 17.24° relative to the 2D plane. The differences in the twisted angles cause different degrees of π cloud overlap along the sp^2^ carbon backbone. The propagation of twisted structures along the backbone decreases the degree of π cloud overlap between neighboring segments and weaken the extension of π conjugation over the 2D skeletons. Therefore, the linker units in the sp^2^ carbon frameworks are a key structural parameter that controls the π conjugation.

### Frontier molecular orbital levels and band structure

From the onset of the electronic absorption band, the optical band gap of sp^2^c-COF was evaluated to be 2.05 eV (Table 2). As a result of decreased π conjugation, sp^2^c-COF-2 exhibited an increased band gap at 2.14 eV while sp^2^c-COF-3 yielded the largest band gap of 2.21 eV.

**Table 2:** Electronic properties

	Reductive potential (V)	Oxidative potential (V)	HOMO (eV)	LUMO (eV)	E_g,opt_ (eV)	E_g,elec_ (eV)
sp^2^c-COF	−0.96	0.94	−5.74	−3.84	2.05	1.90
sp^2^c-COF-2	−0.99	1.08	−5.88	−3.81	2.14	2.07
sp^2^c-COF-3	−1.02	1.1	−5.90	−3.78	2.21	2.12

*E*_*g,opt*_ optical band gap, *E*_*g,elec*_ electrochemical band gap

To investigate the highest occupied molecular orbital (HOMO) and the lowest unoccupied molecular orbital (LUMO) levels of sp^2^ carbon-conjugated COFs, we further conducted cyclic voltammetry of powder samples coated on carbon glass electrode in the presence of tetrabutylammonia hexafulorophosphate electrolyte in acetonitrile at 25 °C (Table 2 and Supplementary Figure 13). sp^2^c-COF exhibited the oxidation and reduction potentials at 0.94 V and –0.96 V, respectively. Associated with the decreased π conjugation, sp^2^c-COF-2 and sp^2^c-COF-3 exhibited the oxidation potentials at 1.08 and 1.10 V, and the reduction potentials at −0.99 and −1.02 V, respectively. From the onset positions of oxidation and reduction potentials, the HOMO and LUMO levels were evaluated to be −5.74 and −5.88 eV for sp^2^c-COF, −5.90, and −3.84 eV for sp^2^c-COF-2 and −3.81 and −3.78 eV for sp^2^c-COF-3, respectively. Owing to the less π conjugation, sp^2^c-COF-2 and sp^2^c-COF-3 exhibited lowered HOMO and higher LUMO levels compared to those of sp^2^c-COF. Clearly, the linker units owing to their twisted conformation, enable the fine regulation of the HOMO and LUMO levels. As calculated from the HOMO and LUMO levels, the electrochemical band gaps of sp^2^c-COF, sp^2^c-COF-2, and sp^2^c-COF-3 were 1.90, 2.07, and 2.12 eV, respectively (Table 2). These results indicate that sp^2^c-COFs are semiconductors with predesigned lattices and tunable bad gap structures.

### Light emitting in solutions

We dispersed sp^2^c-COF, sp^2^c-COF-2, and sp^2^c-COF-3 in different solvents and measured their fluorescence spectra (Supplementary Figures 14–16). sp^2^c-COF in THF emitted a strong red luminescence centered at 616 nm, whereas sp^2^c-COF-2 and sp^2^c-COF-3 emitted orange luminescence centered at 559 and 568 nm, respectively (Supplementary Figure 14g, 15 g and 16 g, Supplementary Table 8). The dispersed samples exhibited linker-dependent emission colors and are totally different from those of monomer and model compound (Fig. 5d). The absolute fluorescence quantum yields of sp^2^c-COF, sp^2^c-COF-2, and sp^2^c-COF-3 were evaluated to be 15%, 19%, and 12%, respectively (Fig. 5e, Supplementary Table 9). In water, sp^2^c-COF, sp^2^c-COF-2, and sp^2^c-COF-3 emitted at 606 nm, 592 nm, and 602 nm, respectively (Supplementary Figures 14a, 15a, 16a, Supplementary Table 8). Notably, their absolute fluorescence quantum yields were as high as 22%, 20%, and 18%, respectively (Fig. 5e, Supplementary Table 9). Moreover, in hexane, sp^2^c-COF, sp^2^c-COF-2, and sp^2^c-COF-3 emitted at 612 nm, 574 nm, and 571 nm (Supplementary Figures 14c, 15c and 16c, Supplementary Table 8) with absolute fluorescence quantum yields of 15%, 14%, and 7%, respectively (Fig. 5e, Supplementary Table 9). In other organic solvents, sp^2^c-COF, sp^2^c-COF-2, and sp^2^c-COF-3 retained fluorescence quantum yields of 15–20% (Fig. 5e, Supplementary Table 9). Fluorescence lifetime measurements revealed that these sp^2^c-COFs dispersed in different solvents have a fluorescence lifetime of 1.3–3.0 ns (Supplementary Table 9, Supplementary Figures 17–19). Therefore, sp^2^c-COFs are highly luminescent and are robust to emit luminescence in various solvents (Fig. 5e, Supplementary Table 9).

### Light emitting in the solid state

The solid samples of sp^2^c-COF, sp^2^c-COF-2, and sp^2^c-COF-3 exhibited electronic absorption bands at 497, 475, and 462 nm, and emitted at 622, 606, and 609 nm, respectively (Fig. 5a–c, red curves, Table 1). The absolute fluorescence quantum yields of the sp^2^c-COF, sp^2^c-COF-2, and sp^2^c-COF-3 solids were 14%, 10%, and 6%, respectively (Fig. 5e, Table 1). Fluorescence microscopy confirmed that sp^2^c-COF is highly emissive across the entire belts (Supplementary Figure 20). The fluorescence lifetime of the solid samples was estimated to be 1.85, 2.93, and 3.43 ns for sp^2^c-COF, sp^2^c-COF-2, and sp^2^c-COF-3, respectively (Fig. 5f). By contrast, the imine-linked pyrene COF did not emit a luminescence in the solid state or when dispersed in organic solvents. Surprisingly, sp^2^c-COF and sp^2^c-COF-2 can keep the emission properties, including fluorescence wavelength, quantum yield and lifetime even upon 1-year exposure to air at room temperature (Fig. 3e, f, Supplementary Figures 7, 8). The combination of stability and light-emitting activity has been long pursued; the sp^2^ carbon frameworks offer a promising platform for designing luminescent and stable materials.

### Light emitting in the exfoliated nanosheets

**Fig. 6:**
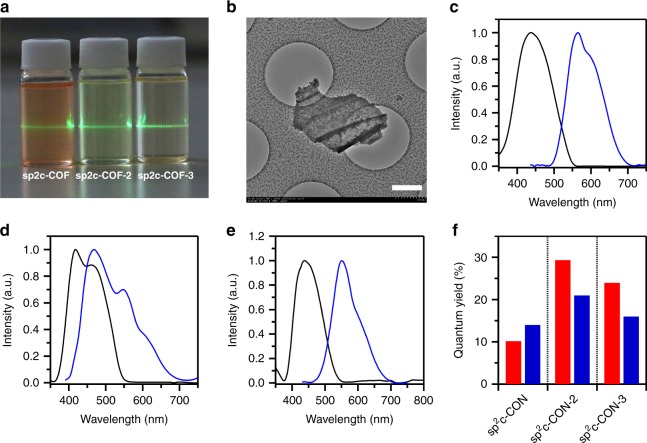
Light-emitting properties of sp^2^c-CONs. **a** Images of Tyndall effect of sp^2^c-CONs after exfoliated in NMP. **b** HR-TEM image of sp^2^c-CON, scale bar 500 nm. **c**–**e** Electronic absorption (black curves) and fluorescence spectra (red curves) of the thin films of **c** sp^2^c-CON, **d** sp^2^c-CON-2, and **e** sp^2^c-CON-3. **f** Absolute fluorescence quantum yields of sp^2^c-CONs in NMP (red bars) and thin films (blue bars)

By virtue of the stable C=C linked 2D skeletons, the 2D sp^2^ carbon covalent organic nanosheets sp^2^c-CON, sp^2^c-CON-2, and sp^2^c-CON-3 were prepared by the sonication-assisted exfoliation of sp^2^c-COF, sp^2^c-COF-2, and sp^2^c-COF-3 in *N*-methyl-2-pyrrolidone (NMP), respectively (ESI). A clear Tyndall effect was observed as a laser light pass through the NMP solutions of the exfoliated sp^2^c-CONs (Fig. 6a).

sp^2^c-CON assumes an ultrathin and transparent sheet structure with crumpled shape as shown by the HR-TEM image (Fig. 6b), which is in contrast to that of sp^2^c-COF with bulk and opaque states. Upon filtration through anodic aluminum oxide (AAO) film, 2D sp^2^c-CON thin films with nearly single layer or limited few layers were prepared (Supplementary Figure 21). Notably, exfoliation induces great change in both absorption and emission. The thin film of sp^2^c-CON exhibited an absorption band at 437 nm (Fig. 6c, black curve) and emitted a luminescence at 565 nm (Fig. 6c, red curve), which are blueshifted from sp^2^c-COF. The thin films of sp^2^c-CON-2 and sp^2^c-CON-3 exhibited absorption bands at 418 and 438 nm (Fig. 6d, e, black curves), and emitted at 468 and 551 nm, respectively (Fig. 6d, e, red curves). The blueshift in the absorption and emission bands suggests that the photochemical events are controlled by the layered and exfoliated sheet status, which are strongly associated with their primary- (nanosheets) and high-order (layer framework) structures. Surprisingly, the sp^2^c-CON-2 and sp^2^c-CON-3 thin films exhibited fluorescence quantum yields of 21% and 16%, respectively (Fig. 6f, blue bars). These absolute quantum yields are 2.1- and 2.7-fold as high as those of sp^2^c-COF-2 and sp^2^c-COF-3, respectively. Moreover, the NMP solutions of exfoliated sp2c-CONs also exhibited high fluorescence quantum yields (Fig. 6f, red bars). Therefore, exfoliation produces brilliant sp^2^ carbon nanosheets.

## Discussion

The above robust light-emitting properties of sp^2^c-COFs and sp^2^c-CONs intrigued us to elucidate the key structural parameters that control the interactions with photons and their photochemical events. In sp^2^c-COFs, pyrene serves as knots to form 2D sp^2^ carbon layer that further stacks to form pyrene columns. Pyrene is a typical chromophore that tends to form excimer once aggregated. For example, boroxine-linked PPy-COF emit a blue luminescence at 484 nm, which is 63-nm redshifted from that (421 nm) of the monomeric pyrene^27^. Since the boroxine linkage does not offer π conjugation along the backbone and the framework does not contain other emissive moieties, the 63-nm redshift suggests that PPy-COF emits an emission from an excimer of pyrene units. The pyrene unit when excited to its singlet state can form an excimer with a ground-state pyrene unit of a neighboring layer to emit the fluorescence. Compared to the excimer emission of pyrene at 484 nm of PPy-COF, sp^2^c-COF emits at a far redshifted wavelength of 622 nm. This 134-nm redshift of emission cannot attribute to only the excimer formation but it is clearly associated with the sp^2^ carbon-conjugated backbone of sp^2^c-COF.

The backbone between two proximate pyrene knots in the same layer consists of the cyano-substituted phenylenevinylene segment, which adopts twisted conformation and is known as aggregation-induced emissive (AIE) chromophore^43^. Together with the excimer formation of the pyrene knots, the AIE feature of the linker backbone reveals a principle that in sp^2^c-COFs, the knot and linker are tightly correlated with the photochemical events, including excitation and emission.

For sp^2^c-COF, the adsorption band is 498 nm for the solid sample, which is blueshifted to 437 nm for the thin film of exfoliated nanosheets. The 61-nm blueshift suggests that in sp^2^c-COF the linkers adopt a more planar conformation so that they can extend the π cloud overlap in a more efficient way. On the other hand, in the exfoliated sp^2^c-CON nanosheets, the π–π stacking force that helps to planarize the sheet is released so that the backbones assume more twisted conformation to cause less π cloud overlap that leads to a blueshift of the absorption band. Indeed, density functional tight binding calculations confirmed that the phenyl twisted angle is 55.61° in the single layer and is 37.81° in the layer framework. Therefore, the photon absorption ruled by Franck-Cotton principle is regulated by the twisted status of the phenylenevinylene backbone. A 61-nm blueshift is observed for the emission band from 626 nm of sp^2^c-COF to 565 nm of the sp^2^c-CON thin film, changing the emission color from red to orange yellow. Notably, the fluorescence quantum yield retained nearly the same level (14% for sp^2^c-COF versus 12% for sp^2^c-CON). This observation suggests that the decreased AIE activity of the linkers in the exfoliated sp^2^c-CON film is balanced by the increased emission activity of the pyrene knots.

Similarly, sp^2^c-COF-2 shows a blueshift of the absorption band from 475 nm of the solid sample to 418 nm of the exfoliated sp^2^c-CON-2 thin film. This result reflects that the biphenyl linker greatly twists so that the transmission of the π conjugation along the *x* and *y* directions is suppressed in the exfoliated nanosheets. Therefore, the pyrene knots become to dominate the photon adsorption in the exfoliated nanosheets. Similarly, such a pyrene-knot dominance is also observable for the emission, which shows a blueshift from 606 (sp^2^c-COF-2) to 468 nm (sp^2^c-CON-2 thin film). Such an interruption of π conjugation enables the generation of a blue emission from the sp^2^c-CON-2 thin film. Interestingly, the fluorescence quantum yield is greatly enhanced from 10% for sp^2^c-COF-2 to 21% for the sp^2^c-CON-2 thin film. This improved activity originates from the enhanced emission from pyrene knots in the exfoliated state.

In the case of sp^2^c-COF-3, the terphenyl linkers also cause the blueshift of the adsorption and emission bands but the degree is the lowest among the series. The blueshift is 24 nm for the absorption band and is 58 nm for the emission band. Therefore, the terphenyl linkers could maintain certain degrees of π conjugation in the exfoliated sp^2^c-CON-3 films. Surprisingly, the fluorescence quantum yield is dramatically increased from the originally 6% of sp^2^c-COF-3 to 16% of the exfoliated sp^2^c-CON-3 film. These observations again indicate that in the exfoliated nanosheets the pyrene knots play a key role in determining the emission quantum yield, while the arylenevinylene linkers control the absorption and emission wavelengths.

As describe above, the sp^2^ carbon-conjugated skeletons offer a platform for designing stable and light-emitting frameworks. The C=C linkage enables the designed synthesis of sp^2^c-COFs and sp^2^c-CONs with different lattice sizes. The π conjugation is controlled by the linker units and their AIE feature is a key to achieve high luminescence quantum yields for the layered frameworks. This result is important for device application in which layer structure forms a basic configuration. The knot units play a key role in producing highly emissive covalent organic nanosheets. Interestingly, the exfoliated nanosheets enhance the fluorescence quantum yield by 2–3-fold compared to the bulk frameworks, leading to the generation of brilliant nanosheets. Clearly, the C=C linkage provides a suitable bridge that enables a strong electronic communication between knots and linkers through which they are cooperative in the photochemical events to achieve exceptional light-emitting activity in both layered frameworks and exfoliated nanosheets.

To ensure the significance of being a 2D structure in achieving the light-emitting function, we synthesized a one-dimensional 1,6-linear pyrene polymer as a control (Supplementary Figure 22a) by condensing (1,6-formylphenyl)pyrene with PDAN to yield a C=C linked sp^2^ carbon polymer that has the similar building units as that of sp^2^c-COF. Compared to sp^2^c-COF, the 1,6-linear pyrene polymer exhibited very weak luminescence in the solid state (Supplementary Figure 22b) and dispersed solutions. For example, the solid sample of 1,6-linear pyrene polymer exhibited the electronic absorption band at 434 nm and emitted at 574 nm (Supplementary Figure 22c); both of them are blueshifted from those of sp^2^c-COF. Moreover, its absolute fluorescence quantum yield is only 1% (Supplementary Figure 22d), which is even one order of magnitude lower than that of sp^2^c-COFs. The fluorescence lifetime is 1.2 ns (Supplementary Figure 22e). These results reflect that the 2D sp^2^ carbon lattice enables far extended π delocalization and greater emission activity and clearly demonstrate that 2D structure is essential for achieving light-emitting functions.

The highly emissive property of sp^2^c-COFs renders them able to detect specific guest molecules that can interact with the skeletons. In sp^2^c-COFs, the polymer backbones possess cyano (–CN) side groups that can serve as a ligand for triggering supramolecular interactions with specific metal species. To prove the concept, we selected Cu^2+^ as a target. Indeed, upon addition of Cu^2+^, the fluorescence intensity of sp^2^c-COF in THF was quenched (Supplementary Figure 23a). By contrast, addition of Zn^2+^ that cannot ligate with the –CN groups could not quench the emission, irrespective of its concentration (Supplementary Figure 23b). These results suggest that interaction with cyano groups is essential for triggering fluorescence sensing. Indeed, sp^2^c-COF is highly sensitive to the Cu^2+^ ion and achieves a detection limit of 88 ppb (ESI). From the Stern–Volmer plot, the fluorescence quenching rate constant *k*_q_ (=*k*_SV_/τ) was calculated to be as high as 4.1 × 10^14^ M^−1^ s^−1^ (Supplementary Figure 23c). The extremely high *k*_q_-value revealed that the sp^2^ carbon-conjugated COF serves as a highly selective and effective sensor for detecting Cu^2+^ ions.

In summary, we have developed a C=C linkage for the designed synthesis of sp^2^ carbon-conjugated covalent organic frameworks. The C=C polycondensation enables the topology-directed connection of pyrene knots and arylenevinylene linkers into predesigned tetragonal sp^2^ carbon lattices that are π conjugated along both the *x* and *y* directions and develop layer frameworks that are stable under various conditions. The sp^2^ carbon frameworks consist of the pyrene knots linked with twisted arylenevinylene backbones so that the photochemical events are highly correlated with their primary- and high-order structures, constituting a class of unique photoactive semiconductors with enriched absorption and emission features. Comparative studies on the bulk frameworks and exfoliated nanosheets disclosed the structural parameters that control the photochemical events. The electronic communication and cooperation between the knots and linkers in the sp^2^ carbon lattices enable the simultaneous realization of highly emissive layered frameworks and exfoliated nanosheets. These characteristics are established in COFs and would exert great impact on the further advance of the field. We envisage that sp^2^ carbon frameworks could offer a promising platform for designing robust photoactive semiconductors that are useful in sensors, energy-harvesting antennae and photocatalysts.

## Methods

### sp^2^c-COF

A 1,4-dioxane (1.0 mL) solution of TFPPy (0.024 mmol, 15.0 mg) and PDAN (0.048 mmol, 7.58 mg) in the presence of KOH catalyst (4 M, 0.1 mL) in a Pyrex tube (10 mL) was degassed via three freeze–pump–thaw cycles. The tube was flame sealed and heated at 90 °C for 3 days. The precipitate was collected via centrifugation, washed three times with H_2_O, five times with THF and then subjected to Soxhlet extraction with THF for 1 day. The powder was collected and dried at 120 °C under vacuum overnight to yield sp^2^c-COF solid in an isolated yield of 84%.

### sp^2^c-COF-2

A 1,4-dioxane (1.0 mL) solution of TFPPy (0.024 mmol, 15.0 mg) and BPDAN (0.048 mmol, 11.26 mg) in the presence of KOH catalyst (4 M, 0.1 mL) in a Pyrex tube (10 mL) was degassed via three freeze–pump–thaw cycles. The tube was sealed by flame and heated at 110 °C for 3 days. The precipitate was collected via centrifugation, washed three times with water, five times with THF and then subjected to Soxhlet extraction with THF for 1 day. The powder was collected and dried at 120 °C under vacuum overnight to yield sp^2^c-COF-2 in an isolated yield of 82%.

### sp^2^c-COF-3

A 1,2-dichlorobenzene (ODCB, 500 μL) solution of TFPPy (0.016 mmol, 10.0 mg) and TPDAN (0.032 mmol, 9.97 mg) in the presence of TBAH catalyst (1 M in MeOH, 500 μL) in a Pyrex tube (10 mL) was degassed via three freeze–pump–thaw cycles. The tube was sealed by flame and heated at 120 °C for 3 days. The precipitate was collected via centrifugation, washed five times with THF and then subjected to Soxhlet extraction with THF for 1 day. The powder was collected and dried at 120 °C under vacuum overnight to yield sp^2^c-COF-3 in an isolated yield of 88%.

### Stability test in solvents

The sp^2^c-COFs samples (15 mg) were kept in 2 mL of DMF, THF, water, MeOH, HCl (12 M) or NaOH (14 M), respectively for 1 week. The samples were washed with THF (for powders treated in organic solvents) or water (for powders treated in acid/base solutions), dried at 120 °C overnight under vacuum and subjected to PXRD, FT IR, and nitrogen sorption isotherm measurements.

### Long-term stability test in air

The sp^2^c-COF and sp^2^c-COF-2 samples were kept in open glass tube and exposed to air for more than 1 year at room temperature. During the period, these samples were not avoided shining sunlight and room lamps. After 1 year, the samples were directly subjected to PXRD, BET, and luminescence measurements without any further treatment.

## Electronic supplementary material

Supplementary Information

## Data Availability

All data generated or analysed during this study are included in this published article (and its supplementary information).
