# Supplementary material for: Designed synthesis of stable light-emitting two-dimensional sp2 carbon-conjugated covalent organic frameworks
Source: Nat Commun. 2018 Oct 8;9:4143. doi: 10.1038/s41467-018-06719-8 (PMC6175883; doi:10.1038/s41467-018-06719-8)
Supplement: Supplementary file 1 — Supplementary Information [file 41467_2018_6719_MOESM1_ESM.pdf]

Supplementary Information

Designed synthesis of stable light-emitting two-dimensional sp^2 carbon-conjugated covalent organic frameworks

Jin, E. *et al.*

Supplementary Table 1 | Atomistic coordinates for the AA-stacking mode of sp^{2}c-COF-1 optimized by using DFTB+ method (space group *P*1*, a* = 34.2613, *b* = 36.6059 Å, *c* = 3.7519 Å, α = 90.0000°, β = 87.5352° and γ = 90.0000°).

Atom	*x/a*	*y/b*	*z/c*
C	0.03620	0.92163	0.47597
C	0.03611	0.96065	0.45733
C	0.07089	0.98127	0.37599
C	0.92790	0.89885	0.52478
C	0.92747	0.86367	0.67986
C	0.89529	0.84052	0.66543
C	0.86113	0.85146	0.49670
C	0.86121	0.88692	0.34700
C	0.89379	0.90968	0.35480
C	0.17422	0.82912	0.52077
C	0.78521	0.77123	0.50325
C	0.74891	0.78753	0.43708
C	0.71478	0.76686	0.43424
C	0.67777	0.70812	0.48781
H	0.70100	0.34460	0.57219
H	0.40196	0.53334	0.69871
H	0.54703	0.64585	0.17849
H	0.60346	0.68633	0.20582
H	0.66472	0.60359	0.78905
H	0.60705	0.56390	0.78378
H	0.74717	0.81701	0.38454
C	0.64337	0.26950	0.45339
N	0.61672	0.25054	0.44003
H	0.68712	0.78055	0.38040
C	0.53620	0.42163	0.47597
C	0.53611	0.46065	0.45733
C	0.57089	0.48127	0.37599
C	0.42790	0.39885	0.52478
C	0.42747	0.36367	0.67986
C	0.39529	0.34052	0.66543
C	0.36113	0.35146	0.49670
C	0.36121	0.38692	0.34700
C	0.39379	0.40968	0.35480
C	0.67422	0.32912	0.52077
C	0.28521	0.27123	0.50325
C	0.24891	0.28753	0.43708
C	0.21478	0.26686	0.43424
C	0.17777	0.20812	0.48781

H	0.20100	0.84460	0.57219
H	0.90196	0.03334	0.69871
H	0.04703	0.14585	0.17849
H	0.10346	0.18633	0.20582
H	0.16472	0.10359	0.78905
H	0.10705	0.06390	0.78378
H	0.24717	0.31701	0.38454
C	0.14337	0.76950	0.45339
N	0.11672	0.75054	0.44003
H	0.18712	0.28055	0.38040
C	0.96380	0.92163	0.52403
C	0.96389	0.96065	0.54267
C	0.92911	0.98127	0.62401
C	0.07210	0.89885	0.47522
C	0.07253	0.86367	0.32014
C	0.10471	0.84052	0.33457
C	0.13887	0.85146	0.50330
C	0.13879	0.88692	0.65300
C	0.10621	0.90968	0.64520
C	0.82578	0.82912	0.47923
C	0.21479	0.77123	0.49675
C	0.25109	0.78753	0.56292
C	0.28522	0.76686	0.56576
C	0.32223	0.70812	0.51219
H	0.29900	0.34460	0.42781
H	0.59804	0.53334	0.30129
H	0.45297	0.64585	0.82151
H	0.39654	0.68633	0.79418
H	0.33528	0.60359	0.21095
H	0.39295	0.56390	0.21622
H	0.25283	0.81701	0.61546
C	0.35663	0.26950	0.54661
N	0.38328	0.25054	0.55997
H	0.31288	0.78055	0.61960
C	0.46380	0.42163	0.52403
C	0.46389	0.46065	0.54267
C	0.42911	0.48127	0.62401
C	0.57210	0.39885	0.47522
C	0.57253	0.36367	0.32014
C	0.60471	0.34052	0.33457
C	0.63887	0.35146	0.50330
C	0.63879	0.38692	0.65300
C	0.60621	0.40968	0.64520
C	0.32578	0.32912	0.47923

C	0.71479	0.27123	0.49675
C	0.75109	0.28753	0.56292
C	0.78522	0.26686	0.56576
C	0.82223	0.20812	0.51219
H	0.79900	0.84460	0.42781
H	0.09804	0.03334	0.30129
H	0.95297	0.14585	0.82151
H	0.89654	0.18633	0.79418
H	0.83528	0.10359	0.21095
H	0.89295	0.06390	0.21622
H	0.75283	0.31701	0.61546
C	0.85663	0.76950	0.54661
N	0.88328	0.75054	0.55997
H	0.81288	0.28055	0.61960
C	0.96380	0.07837	0.52403
C	0.96389	0.03935	0.54267
C	0.92911	0.01873	0.62401
C	0.07210	0.10115	0.47522
C	0.07253	0.13633	0.32014
C	0.10471	0.15948	0.33457
C	0.13887	0.14854	0.50330
C	0.13879	0.11308	0.65300
C	0.10621	0.09032	0.64520
C	0.82578	0.17088	0.47923
C	0.21479	0.22877	0.49675
C	0.25109	0.21247	0.56292
C	0.28522	0.23314	0.56576
C	0.32223	0.29188	0.51219
H	0.29900	0.65540	0.42781
H	0.59804	0.46666	0.30129
H	0.45297	0.35415	0.82151
H	0.39654	0.31367	0.79418
H	0.33528	0.39641	0.21095
H	0.39295	0.43610	0.21622
H	0.25283	0.18299	0.61546
C	0.35663	0.73050	0.54661
N	0.38328	0.74946	0.55997
H	0.31288	0.21945	0.61960
C	0.46380	0.57837	0.52403
C	0.46389	0.53935	0.54267
C	0.42911	0.51873	0.62401
C	0.57210	0.60115	0.47522
C	0.57253	0.63633	0.32014
C	0.60471	0.65948	0.33457

C	0.63887	0.64854	0.50330
C	0.63879	0.61308	0.65300
C	0.60621	0.59032	0.64520
C	0.32578	0.67088	0.47923
C	0.71479	0.72877	0.49675
C	0.75109	0.71247	0.56292
C	0.78522	0.73314	0.56576
C	0.82223	0.79188	0.51219
H	0.79900	0.15540	0.42781
H	0.09804	0.96666	0.30129
H	0.95297	0.85415	0.82151
H	0.89654	0.81367	0.79418
H	0.83528	0.89641	0.21095
H	0.89295	0.93610	0.21622
H	0.75283	0.68299	0.61546
C	0.85663	0.23050	0.54661
N	0.88328	0.24946	0.55997
H	0.81288	0.71945	0.61960
C	0.03620	0.07837	0.47597
C	0.03611	0.03935	0.45733
C	0.07089	0.01873	0.37599
C	0.92790	0.10115	0.52478
C	0.92747	0.13633	0.67986
C	0.89529	0.15948	0.66543
C	0.86113	0.14854	0.49670
C	0.86121	0.11308	0.34700
C	0.89379	0.09032	0.35480
C	0.17422	0.17088	0.52077
C	0.78521	0.22877	0.50325
C	0.74891	0.21247	0.43708
C	0.71478	0.23314	0.43424
C	0.67777	0.29188	0.48781
H	0.70100	0.65540	0.57219
H	0.40196	0.46666	0.69871
H	0.54703	0.35415	0.17849
H	0.60346	0.31367	0.20582
H	0.66472	0.39641	0.78905
H	0.60705	0.43610	0.78378
H	0.74717	0.18299	0.38454
C	0.64337	0.73050	0.45339
N	0.61672	0.74946	0.44003
H	0.68712	0.21945	0.38040
C	0.53620	0.57837	0.47597
C	0.53611	0.53935	0.45733

C	0.57089	0.51873	0.37599
C	0.42790	0.60115	0.52478
C	0.42747	0.63633	0.67986
C	0.39529	0.65948	0.66543
C	0.36113	0.64854	0.49670
C	0.36121	0.61308	0.34700
C	0.39379	0.59032	0.35480
C	0.67422	0.67088	0.52077
C	0.28521	0.72877	0.50325
C	0.24891	0.71247	0.43708
C	0.21478	0.73314	0.43424
C	0.17777	0.79188	0.48781
H	0.20100	0.15540	0.57219
H	0.90196	0.96666	0.69871
H	0.04703	0.85415	0.17849
H	0.10346	0.81367	0.20582
H	0.16472	0.89641	0.78905
H	0.10705	0.93610	0.78378
H	0.24717	0.68299	0.38454
C	0.14337	0.23050	0.45339
N	0.11672	0.24946	0.44003
H	0.18712	0.71945	0.38040
C	0.50000	0.48030	0.50000
C	0.50000	0.40351	0.50000
H	0.50000	0.62651	0.50000
C	-0.00000	0.98030	0.50000
C	-0.00000	0.90351	0.50000
H	-0.00000	0.12651	0.50000
C	0.50000	0.51970	0.50000
C	0.50000	0.59649	0.50000
H	0.50000	0.37349	0.50000
C	-0.00000	0.01970	0.50000
C	0.00000	0.09649	0.50000
H	0.00000	0.87349	0.50000

**Supplementary Table 2 | ** Atomistic coordinates for the refined unit cell parameters for sp[2]c-COF via Pawley refinement (space group *C2/M*, a = 33.9309 Å, b = 34.8219 Å, c = 3.7676 Å, α = 90.0000°, β = 106.6756° and γ = 90.00000°).

Atom	*x/a*	*y/b*	*z/c*
C	0.03469	0.91868	0.46850
C	0.03406	0.95929	0.44396
C	0.06550	0.98038	0.34460
C	0.92865	0.89666	0.52856
C	0.93224	0.86082	0.70625
C	0.89774	0.83900	0.68862
C	0.85893	0.85288	0.49328
C	0.85516	0.88906	0.32102
C	0.88983	0.91045	0.33276
C	0.17755	0.83057	0.52948
C	0.78449	0.77039	0.47477
C	0.74607	0.78851	0.38987
C	0.71191	0.76836	0.41608
C	0.67873	0.70851	0.53246
H	0.70580	0.34665	0.54751
H	0.41084	0.53526	0.74405
H	0.53754	0.64969	0.13584
H	0.59939	0.68855	0.16535
H	0.67519	0.60041	0.83339
H	0.61293	0.56237	0.82021
H	0.74268	0.81850	0.29730
C	0.64397	0.26860	0.54956
N	0.61839	0.24899	0.58967
H	0.68181	0.78223	0.34595
C	0.50000	0.47957	0.50000
C	0.50000	0.39908	0.50000
H	0.50000	0.63245	0.50000
C	0.03469	0.91868	0.46850
C	0.03406	0.95929	0.44396
C	0.06550	0.98038	0.34460
C	0.92865	0.89666	0.52856
C	0.93224	0.86082	0.70625
C	0.89774	0.83900	0.68862
C	0.85893	0.85288	0.49328
C	0.85516	0.88906	0.32102
C	0.88983	0.91045	0.33276
C	0.17755	0.83057	0.52948
C	0.78449	0.77039	0.47477

C	0.74607	0.78851	0.38987
C	0.71191	0.76836	0.41608
C	0.67873	0.70851	0.53246
H	0.70580	0.34665	0.54751
H	0.41084	0.53526	0.74405
H	0.53754	0.64969	0.13584
H	0.59939	0.68855	0.16535
H	0.67519	0.60041	0.83339
H	0.61293	0.56237	0.82021
H	0.74268	0.81850	0.29730
C	0.64397	0.26860	0.54956
N	0.61839	0.24899	0.58967
H	0.68181	0.78223	0.34595
C	0.50000	0.47957	0.50000
C	0.50000	0.39908	0.50000
H	0.50000	0.63245	0.50000

Supplementary Table 3 | Atomistic coordinates for the AA-stacking mode of sp^{2}c-COF-2 optimized by using DFTB+ method (space group *P*1*,* a = 28.9934 Å, b = 28.9393Å, c = 3.7367 Å, α = 89.5259°, β = 90.007° and γ = 89.6156°).

Atom	*x/a*	*y/b*	*z/c*
C	0.27656	0.05267	1.81606
C	0.29034	0.09909	1.8087
C	0.25522	0.13404	1.80936
C	0.20754	0.12123	1.83338
C	0.19522	0.07323	1.84774
C	0.23043	0.03845	1.83678
C	0.26586	0.18229	1.79328
C	0.17214	0.15654	1.85052
C	0.18466	0.20452	1.86348
C	0.2327	0.21555	1.8406
C	0.14945	0.23928	1.88992
C	0.10331	0.22472	1.89234
C	0.08944	0.17839	1.88318
C	0.12443	0.14348	1.8614
C	0.11363	0.09522	1.85538
C	0.14727	0.0619	1.86974
H	0.13651	0.02571	1.89384
H	0.07759	0.08388	1.84534
H	0.30143	0.19368	1.7463
H	0.30355	0.0258	1.81082
H	0.24412	0.25153	1.8539
H	0.07612	0.2513	1.90676
C	0.34074	0.10776	1.8212
C	0.36028	0.1448	0.01214
C	0.37178	0.07603	1.66188
C	0.4078	0.15092	0.03228
H	0.33833	0.16844	0.1667
C	0.41929	0.0809	1.69504
H	0.3588	0.04716	1.50132
C	0.43865	0.11885	1.87686
H	0.42084	0.17986	0.19032
H	0.44214	0.0552	1.56862
C	0.22271	0.98782	1.86492
C	0.18582	0.96412	1.70244
C	0.25523	0.96079	0.05804
C	0.18072	0.91657	1.74776
H	0.16157	0.98231	1.5232
C	0.25128	0.91311	0.0913

H	0.28397	0.9775	0.198
C	0.21333	0.88972	1.94162
H	0.15135	0.89941	1.6183
H	0.27726	0.89431	0.25008
C	0.03902	0.17004	1.90876
C	0.0196	0.13309	0.10584
C	0.00794	0.20211	1.75008
C	0.97205	0.12737	0.13246
H	0.0417	0.1091	0.259
C	0.96047	0.19757	1.78874
H	0.02087	0.23088	1.58452
C	0.94114	0.15973	1.97704
H	0.95911	0.09828	0.29304
H	0.9376	0.22345	1.66152
C	0.15652	0.28992	1.92788
C	0.12387	0.31539	0.12958
C	0.19237	0.31554	1.76546
C	0.12636	0.36306	0.16732
H	0.09593	0.29745	0.27232
C	0.19638	0.36297	1.81832
H	0.21661	0.29931	1.57794
C	0.16336	0.38819	0.01716
H	0.10005	0.38042	0.3304
H	0.22503	0.38145	1.68826
C	0.17011	0.43778	0.0604
H	0.20374	0.45052	1.96354
C	0.48875	0.12088	1.91148
H	0.50698	0.08879	1.83548
C	0.14046	0.47052	0.19276
C	0.5159	0.15673	0.02038
C	0.15369	0.51988	0.21112
C	0.1218	0.55468	0.11106
C	0.19804	0.53323	0.32386
C	0.13451	0.60105	0.11006
H	0.08699	0.54514	0.02222
C	0.21045	0.57969	0.32688
H	0.22312	0.50702	0.4172
C	0.17919	0.61447	0.2145
H	0.10936	0.62717	0.01698
H	0.24499	0.58898	0.424
C	0.5666	0.1517	0.05478
C	0.5959	0.18869	1.96328
C	0.58682	0.11017	0.18092
C	0.64365	0.18381	1.98804

H	0.58114	0.22143	1.86278
C	0.63459	0.10549	0.20774
H	0.56493	0.0812	0.26754
C	0.66399	0.14208	0.10884
H	0.66553	0.21287	1.9036
H	0.64907	0.07294	0.31482
C	0.2343	0.80666	0.11888
C	0.20543	0.84019	1.98354
H	0.17155	0.82836	1.8865
C	0.86347	0.12292	0.1333
C	0.89098	0.15781	0.00836
H	0.87287	0.18901	1.90776
C	0.4979	0.20225	0.09848
N	0.48614	0.23927	0.16448
C	0.88079	0.07865	0.25682
N	0.89157	0.04282	0.36492
C	0.09404	0.46088	0.3106
N	0.05695	0.45636	0.41414
C	0.28084	0.81506	0.23922
N	0.31787	0.81833	0.34664
C	0.71487	0.13685	0.12692
C	0.73661	0.09536	0.0219
C	0.74291	0.17339	0.24426
C	0.78449	0.09068	0.03008
H	0.71588	0.0664	1.923
C	0.79083	0.16899	0.2494
H	0.72712	0.2057	0.34006
C	0.81246	0.1277	0.13898
H	0.80051	0.05821	1.94068
H	0.81163	0.19791	0.3484
C	0.19322	0.66362	0.1986
C	0.23792	0.67582	0.084
C	0.16229	0.69943	0.29078
C	0.25124	0.72193	0.06152
H	0.26266	0.64882	0.0034
C	0.17533	0.74563	0.26462
H	0.12761	0.69126	0.39184
C	0.21995	0.75767	0.1477
H	0.28612	0.73043	1.9669
H	0.15064	0.77273	0.34442

Supplementary Table 4 | Atomistic coordinates for the refined unit cell parameters for sp[2]c-COF-2 via Pawley refinement (space group *P*1*,* a = 29.1468 Å, b = 28.6019Å, c = 3.7293 Å, α = 89.7834°, β = 90.2665°and γ = 89.0697°).

Atom	*x/a*	*y/b*	*z/c*
C	0.27656	0.05267	1.81606
C	0.29034	0.09909	1.8087
C	0.25522	0.13404	1.80936
C	0.20754	0.12123	1.83338
C	0.19522	0.07323	1.84774
C	0.23043	0.03845	1.83678
C	0.26586	0.18229	1.79328
C	0.17214	0.15654	1.85052
C	0.18466	0.20452	1.86348
C	0.2327	0.21555	1.8406
C	0.14945	0.23928	1.88992
C	0.10331	0.22472	1.89234
C	0.08944	0.17839	1.88318
C	0.12443	0.14348	1.8614
C	0.11363	0.09522	1.85538
C	0.14727	0.0619	1.86974
H	0.13651	0.02571	1.89384
H	0.07759	0.08388	1.84534
H	0.30143	0.19368	1.7463
H	0.30355	0.0258	1.81082
H	0.24412	0.25153	1.8539
H	0.07612	0.2513	1.90676
C	0.34074	0.10776	1.8212
C	0.36028	0.1448	0.01214
C	0.37178	0.07603	1.66188
C	0.4078	0.15092	0.03228
H	0.33833	0.16844	0.1667
C	0.41929	0.0809	1.69504
H	0.3588	0.04716	1.50132
C	0.43865	0.11885	1.87686
H	0.42084	0.17986	0.19032
H	0.44214	0.0552	1.56862
C	0.22271	0.98782	1.86492
C	0.18582	0.96412	1.70244
C	0.25523	0.96079	0.05804
C	0.18072	0.91657	1.74776
H	0.16157	0.98231	1.5232
C	0.25128	0.91311	0.0913
H	0.28397	0.9775	0.198
C	0.21333	0.88972	1.94162

H	0.15135	0.89941	1.6183
H	0.27726	0.89431	0.25008
C	0.03902	0.17004	1.90876
C	0.0196	0.13309	0.10584
C	0.00794	0.20211	1.75008
C	0.97205	0.12737	0.13246
H	0.0417	0.1091	0.259
C	0.96047	0.19757	1.78874
H	0.02087	0.23088	1.58452
C	0.94114	0.15973	1.97704
H	0.95911	0.09828	0.29304
H	0.9376	0.22345	1.66152
C	0.15652	0.28992	1.92788
C	0.12387	0.31539	0.12958
C	0.19237	0.31554	1.76546
C	0.12636	0.36306	0.16732
H	0.09593	0.29745	0.27232
C	0.19638	0.36297	1.81832
H	0.21661	0.29931	1.57794
C	0.16336	0.38819	0.01716
H	0.10005	0.38042	0.3304
H	0.22503	0.38145	1.68826
C	0.17011	0.43778	0.0604
H	0.20374	0.45052	1.96354
C	0.48875	0.12088	1.91148
H	0.50698	0.08879	1.83548
C	0.14046	0.47052	0.19276
C	0.5159	0.15673	0.02038
C	0.15369	0.51988	0.21112
C	0.1218	0.55468	0.11106
C	0.19804	0.53323	0.32386
C	0.13451	0.60105	0.11006
H	0.08699	0.54514	0.02222
C	0.21045	0.57969	0.32688
H	0.22312	0.50702	0.4172
C	0.17919	0.61447	0.2145
H	0.10936	0.62717	0.01698
H	0.24499	0.58898	0.424
C	0.5666	0.1517	0.05478
C	0.5959	0.18869	1.96328
C	0.58682	0.11017	0.18092
C	0.64365	0.18381	1.98804
H	0.58114	0.22143	1.86278
C	0.63459	0.10549	0.20774

H	0.56493	0.0812	0.26754
C	0.66399	0.14208	0.10884
H	0.66553	0.21287	1.9036
H	0.64907	0.07294	0.31482
C	0.2343	0.80666	0.11888
C	0.20543	0.84019	1.98354
H	0.17155	0.82836	1.8865
C	0.86347	0.12292	0.1333
C	0.89098	0.15781	0.00836
H	0.87287	0.18901	1.90776
C	0.4979	0.20225	0.09848
N	0.48614	0.23927	0.16448
C	0.88079	0.07865	0.25682
N	0.89157	0.04282	0.36492
C	0.09404	0.46088	0.3106
N	0.05695	0.45636	0.41414
C	0.28084	0.81506	0.23922
N	0.31787	0.81833	0.34664
C	0.71487	0.13685	0.12692
C	0.73661	0.09536	0.0219
C	0.74291	0.17339	0.24426
C	0.78449	0.09068	0.03008
H	0.71588	0.0664	1.923
C	0.79083	0.16899	0.2494
H	0.72712	0.2057	0.34006
C	0.81246	0.1277	0.13898
H	0.80051	0.05821	1.94068
H	0.81163	0.19791	0.3484
C	0.19322	0.66362	0.1986
C	0.23792	0.67582	0.084
C	0.16229	0.69943	0.29078
C	0.25124	0.72193	0.06152
H	0.26266	0.64882	0.0034
C	0.17533	0.74563	0.26462
H	0.12761	0.69126	0.39184
C	0.21995	0.75767	0.1477
H	0.28612	0.73043	1.9669
H	0.15064	0.77273	0.34442

Supplementary Table 5 | Atomistic coordinates for the AA-stacking mode of sp^{2}c-COF-3 optimized by using DFTB+ method (space group *C2/M*, a = 48.9627 Å, b = 45.8166 Å, c = 3.6791 Å, α = 90.0000°, β = 103.0499° and γ = 90.0000°)

Atom	*x/a*	*y/b*	*z/c*
C	-0.17674	0.83171	0.47787
C	-0.20093	0.81522	0.46193
H	-0.35815	0.37532	0.61560
H	-0.37929	0.58431	0.83057
H	-0.41976	0.55246	0.83048
H	-0.17884	0.85528	0.42188
H	-0.22136	0.82632	0.39095
C	-0.47524	0.43751	0.47478
C	-0.47553	0.46864	0.45916
C	-0.45258	0.48504	0.38180
C	0.09853	0.81782	0.28196
N	0.07948	0.80278	0.18435
C	0.04983	0.91949	0.46872
C	0.06967	0.87417	0.28443
C	0.09689	0.88185	0.48335
C	-0.12190	0.86393	0.49706
H	-0.43492	0.52672	0.31088
C	-0.40014	0.40903	0.67058
C	-0.42298	0.42727	0.66625
C	-0.19974	0.21512	0.52988
C	-0.14875	0.21164	0.63435
C	-0.12358	0.16491	0.59125
H	-0.06621	0.14630	0.87336
C	-0.04691	0.10748	0.72125
C	0.22531	0.76723	0.48656
C	0.25041	0.77985	0.43442
H	0.25114	0.80333	0.37628
C	-0.22562	0.73690	0.44751
H	-0.20674	0.72604	0.39911
C	0.14993	0.18130	0.43500
C	0.67293	0.72807	0.38385
H	0.32919	0.75156	0.67618
H	1.52615	1.61418	0.11678
H	1.62827	1.72258	0.29551
C	0.00000	0.01571	0.50000
C	0.50000	0.57706	0.50000
H	0.50000	0.60106	0.50000

**Supplementary Table 6 | ** Atomistic coordinates for the refined unit cell parameters for sp^{2}c-COF-3 via Pawley refinement (space group *C2/M*, a = 48.8867 Å, b = 45.8095 Å, c = 3.6802 Å, α = 90.0000°, β = 103.0632° and γ = 90.0000°).

Atom	*x/a*	*y/b*	*z/c*
C	-0.17674	0.83171	0.47787
C	-0.20093	0.81522	0.46193
H	-0.35815	0.37532	0.61560
H	-0.37929	0.58431	0.83057
H	-0.41976	0.55246	0.83048
H	-0.17884	0.85528	0.42188
H	-0.22136	0.82632	0.39095
C	-0.47524	0.43751	0.47478
C	-0.47553	0.46864	0.45916
C	-0.45258	0.48504	0.38180
C	0.09853	0.81782	0.28196
N	0.07948	0.80278	0.18435
C	0.04983	0.91949	0.46872
C	0.06967	0.87417	0.28443
C	0.09689	0.88185	0.48335
C	-0.12190	0.86393	0.49706
H	-0.43492	0.52672	0.31088
C	-0.40014	0.40903	0.67058
C	-0.42298	0.42727	0.66625
C	-0.19974	0.21512	0.52988
C	-0.14875	0.21164	0.63435
C	-0.12358	0.16491	0.59125
H	-0.06621	0.14630	0.87336
C	-0.04691	0.10748	0.72125
C	0.22531	0.76723	0.48656
C	0.25041	0.77985	0.43442
H	0.25114	0.80333	0.37628
C	-0.22562	0.73690	0.44751
H	-0.20674	0.72604	0.39911
C	0.14993	0.18130	0.43500
C	0.67293	0.72807	0.38385
H	0.32919	0.75156	0.67618
H	1.52615	1.61418	0.11678
H	1.62827	1.72258	0.29551
C	0.00000	0.01571	0.50000
C	0.50000	0.57706	0.50000
H	0.50000	0.60106	0.50000

**Supplementary Table 7 | ** Elemental analysis of sp^2c-COFs.

COFs		C (%)	H (%)	N (%)
sp^2c-COF	Calcd.	89.49	3.99	6.52
	Found	87.33	4.21	6.19
sp^2c-COF-2	Calcd.	90.26	4.16	5.54
	Found	89.14	4.59	5.15
sp^2c-COF-3	Calcd.	90.85	4.33	4.82
	Found	88.63	4.74	5.07

**Supplementary Table 8 | ** Luminescence peak of sp^2c-COFs dispersed in different solvents.

	Emission band (nm)						
	THF	CH_2Cl_2	toluene	CH_3CN	MeOH	hexane	H_2O
sp^2c-COF	616	613	612	607	610	612	606
sp^2c-COF-2	559	568	568	575	564	574	592
sp^2c-COF-3	568	568	566	608	567	571	602

**Supplementary Table 9 | ** Fluorescence quantum yield and lifetime of sp^{2}c-COFs dispersed in different solvents.

	Different solvents													
	THF		CH_2Cl_2		Toluene		CH_3CN		MeOH		Hexane		H_2O	
	QY[a] (%)	LT[b] (ns)	QY (%)	LT (ns)	QY (%)	LF (ns)	QY (%)	LT (ns)	QY (%)	LT (ns)	QY (%)	LT (ns)	QY (%)	LT (ns)
sp^{2}c-COF	15	1.1	13	0.8	19	1.8	17	1.0	16	1.2	15	1.8	22	3.2
sp^{2}c-COF-2	19	2.5	16	1.6	21	2.8	13	1.3	18	1.7	14	2.6	20	1.4
sp^{2}c-COF-3	12	2.1	13	1.3	17	2.5	9	1.3	12	1.8	7	3.0	18	1.4

[a]QY = Absolute fluorescence quantum yield. [b]LT = Fluorescence lifetime.

Supplementary Figures

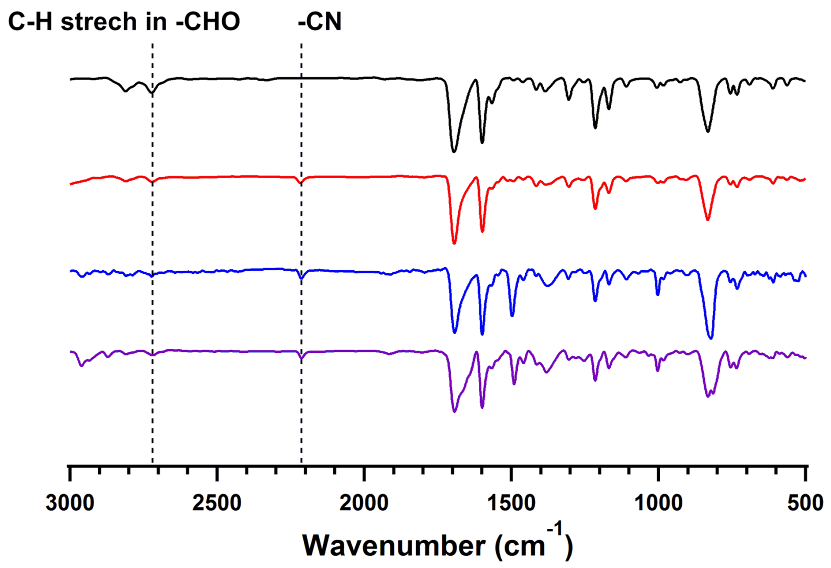

Supplementary Figure 1 | FT IR spectra. FT IR spectra of TFPPy monomer (black curve), sp^2c-COF (red curve), sp^2c-COF-2 (blue curve) and sp^2c-COF-3 (purple curve).

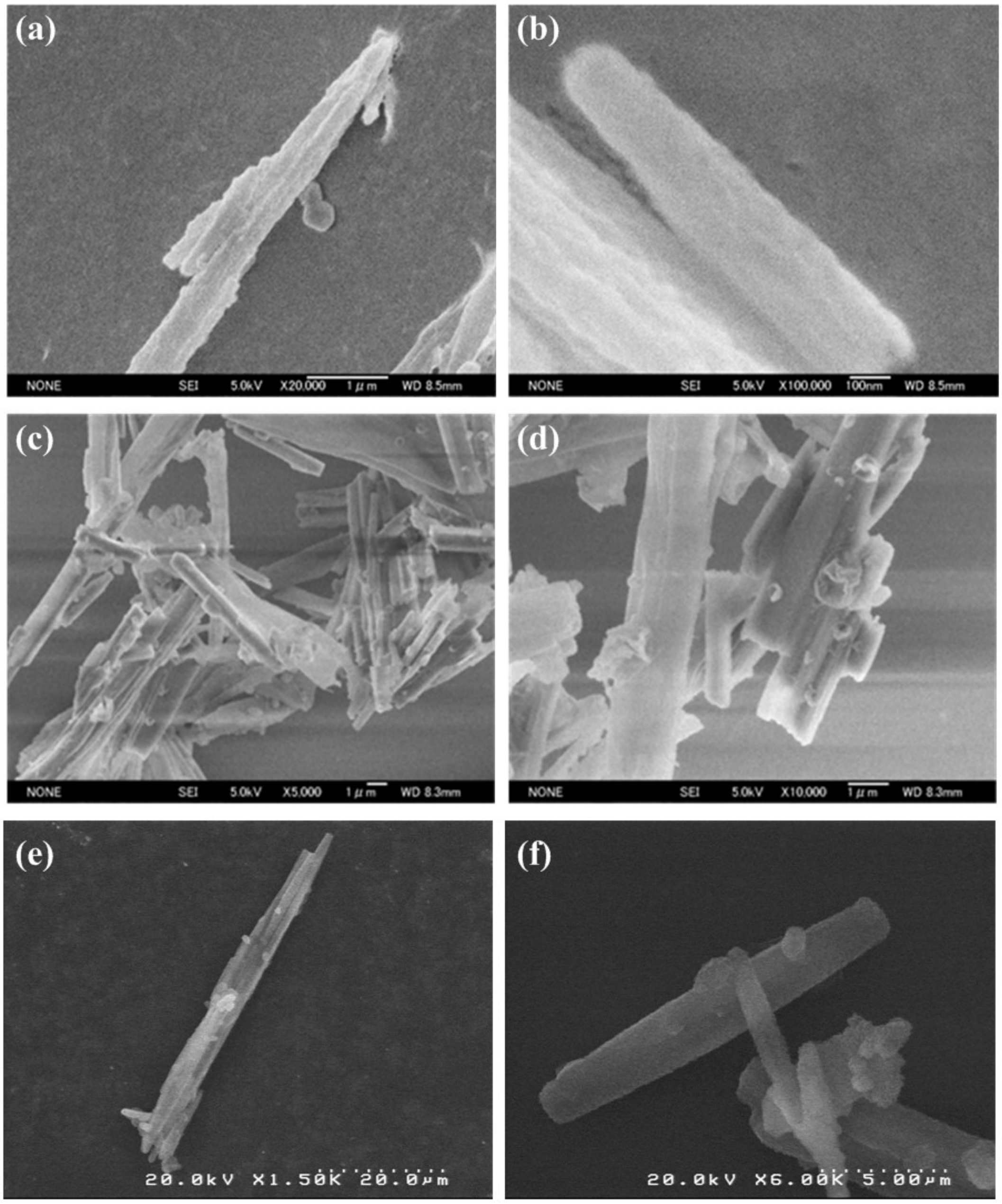

Supplementary Figure 2 | FE-SEM images. FE-SEM images of (a and b) sp^{2}c-COF, (c and d) sp^{2}c-COF-2 and (e and f) sp^{2}c-COF-3.

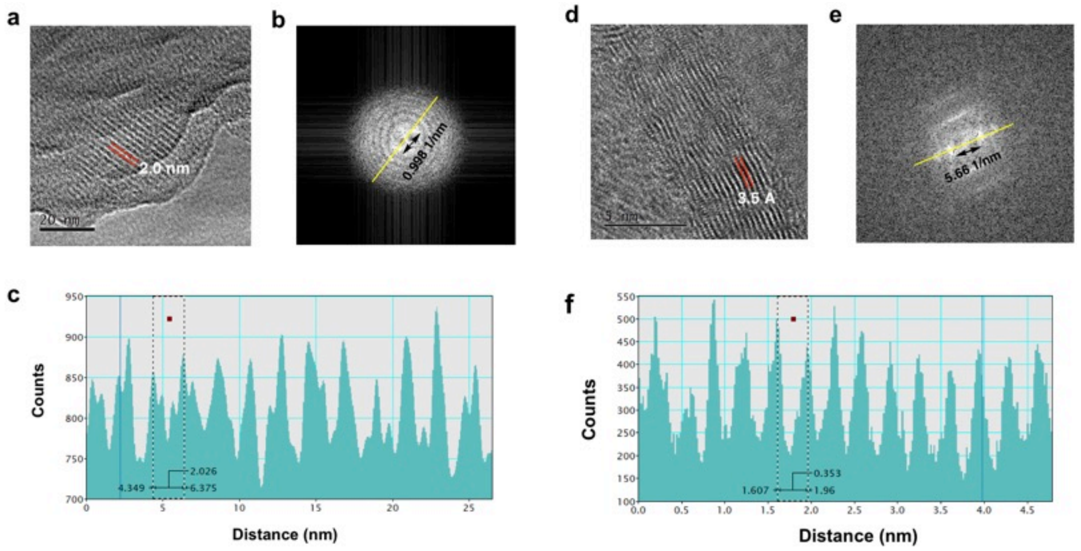

Supplementary Figure 3 | HR TEM images. (a) HR TEM image of sp^2c-COF with pore size of 2 nm, (b) corresponding FFT and (c) line profile along the indicated area in TEM image. (d) HR TEM image of sp^2c-COF with layer to layer distance about 3.5 Å, (e) corresponding FFT and (f) line profile along the indicated area in TEM image.

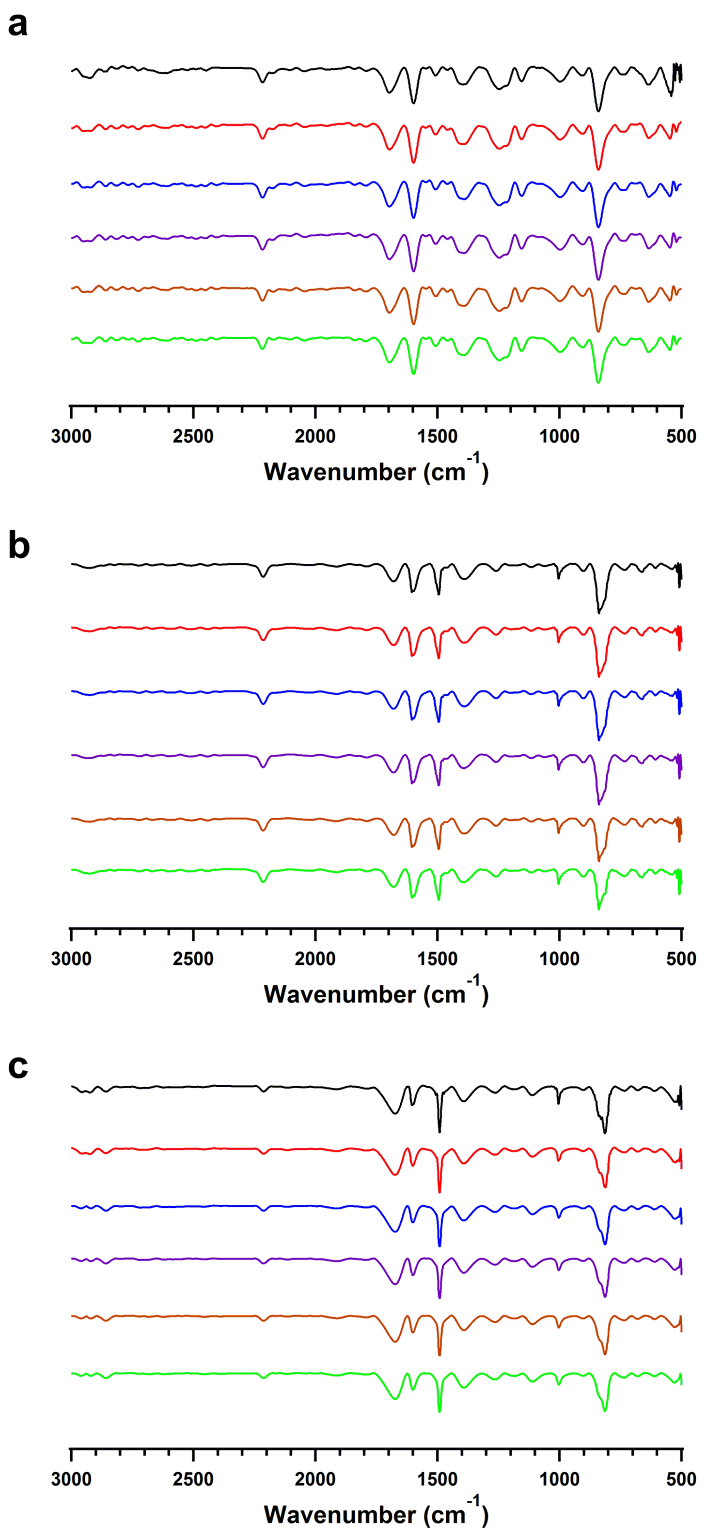

Supplementary Figure 4 | Stability in different solvents for 1 week. FT IR spectra of sp^{2}c-COF, sp^{2}c-COF-2 and sp^{2}c-COF-3 after dispersed in different solvents in different solvents, including DMF (black curve), THF (red curve), MeOH (blue curve), H_2O (purple curve), aqueous KOH solution (14 M, orange curve) and concentrated HCl solution (12 M, green curve).

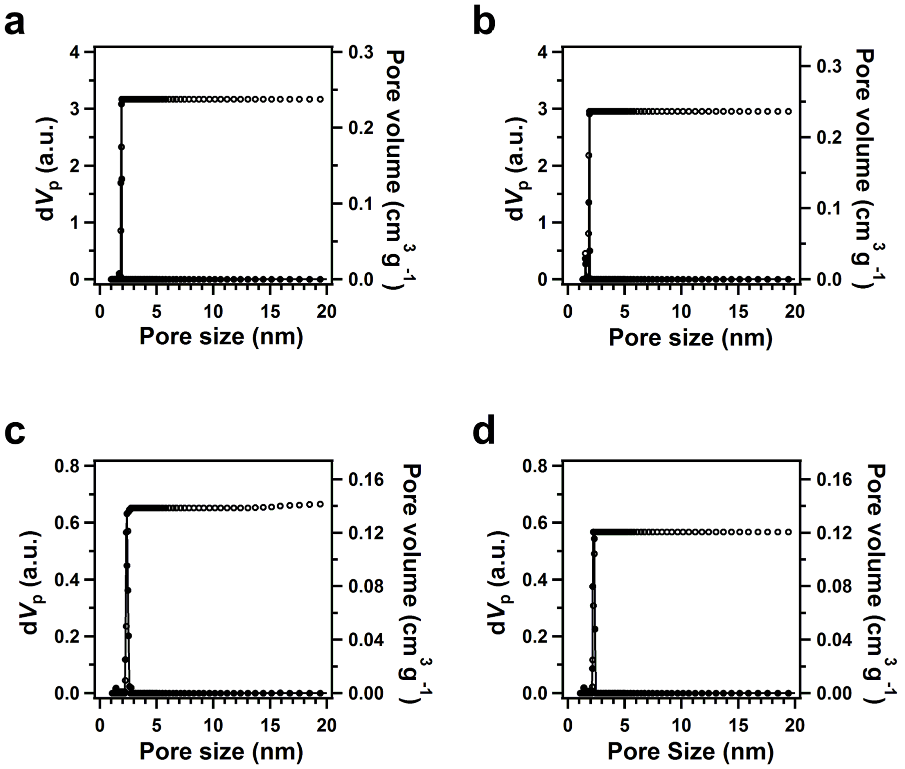

Supplementary Figure 5 | Long-term stability upon exposure to air. Pore-size distribution profiles of sp^{2}c-COF before (a) and after (b) exposed to air for 1 year, and sp^{2}c-COF-2 before (c) and after (d) exposed to air for 1 year.

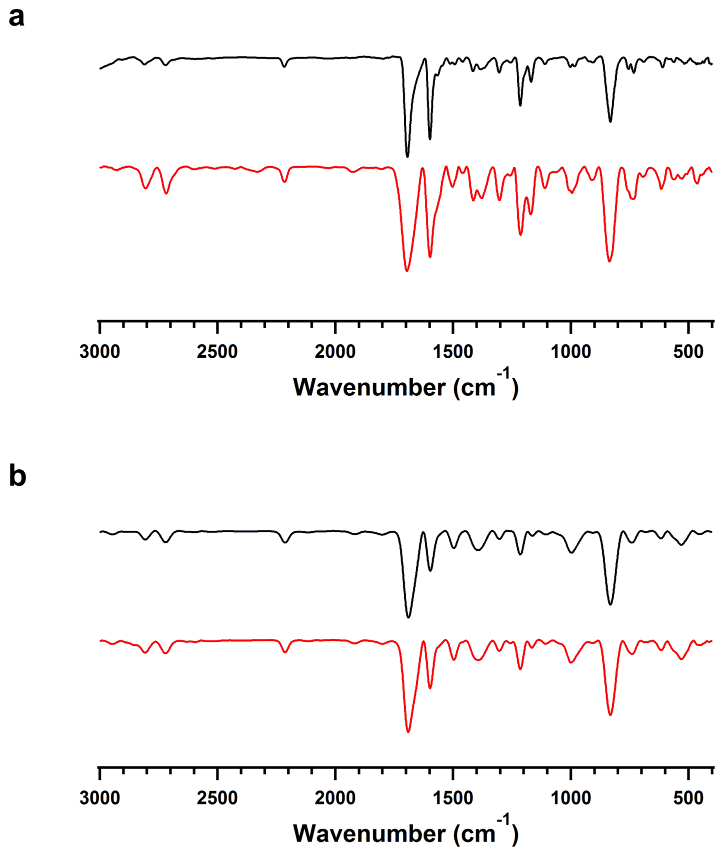

Supplementary Figure 6 | Long-term stability upon exposure to air. FT IR spectra of (a) sp^2c-COF before (black curve) and after (red curve) exposed to air for 1 year and (b) sp^2c-COF-2 before (black curve) and after (red curve) exposed to air for 1 year.

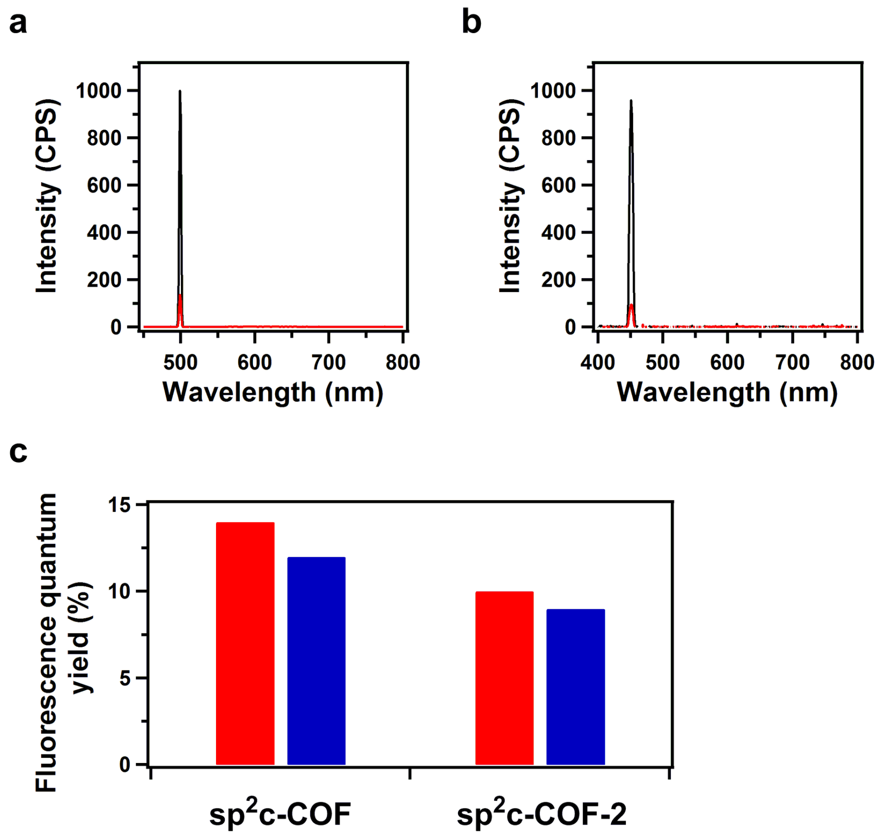

Supplementary Figure 7 | Long-term stability upon exposure to air. **a, b,** Spectra of solid-state absolute quantum yield of (a) sp^{2}c-COF and (b) sp^{2}c-COF-2 after 1-year exposure to air, measured using integral sphere. **c,** Absolute fluorescence quantum yield of sp^{2}c-COF and sp^{2}c-COF-2 of as-synthesized samples (red bars) and samples after 1-year exposure to air (blue bars).

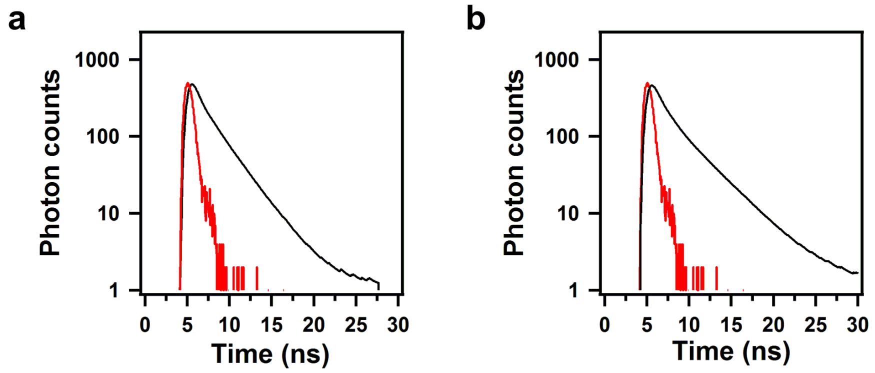

Supplementary Figure 8 | Long-term stability upon exposure to air. Solid-state fluorescence lifetime spectra (black curves) of (a) sp^2c-COF and (b) sp^2c-COF-2 exposed to air for 1 year. The red curve is the instrumental response file.

a

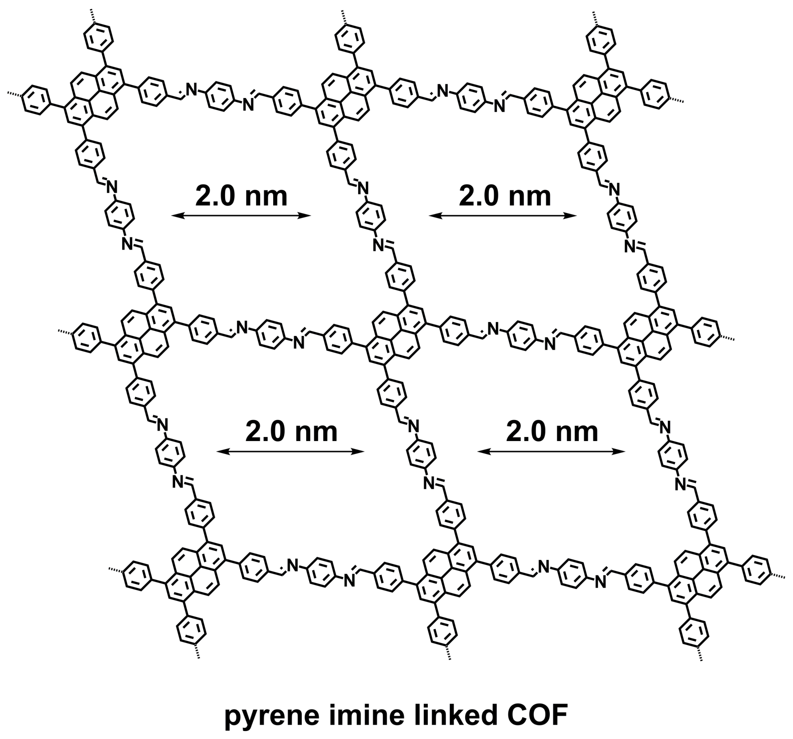

b

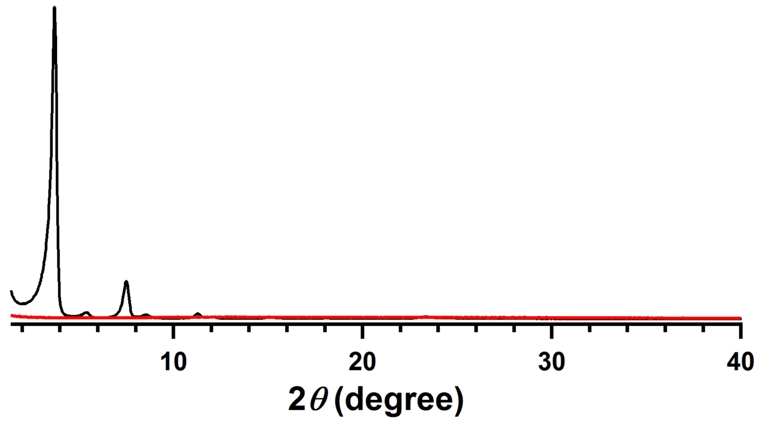

Supplementary Figure 9 | (a) Chemical Structure of an imine-linked pyrene COF with similar skeleton to sp^2c-COF. (b) PXRD patterns of the imine-linked pyrene COF before (black curve) and after (red curve) exposed to air for 1 year.

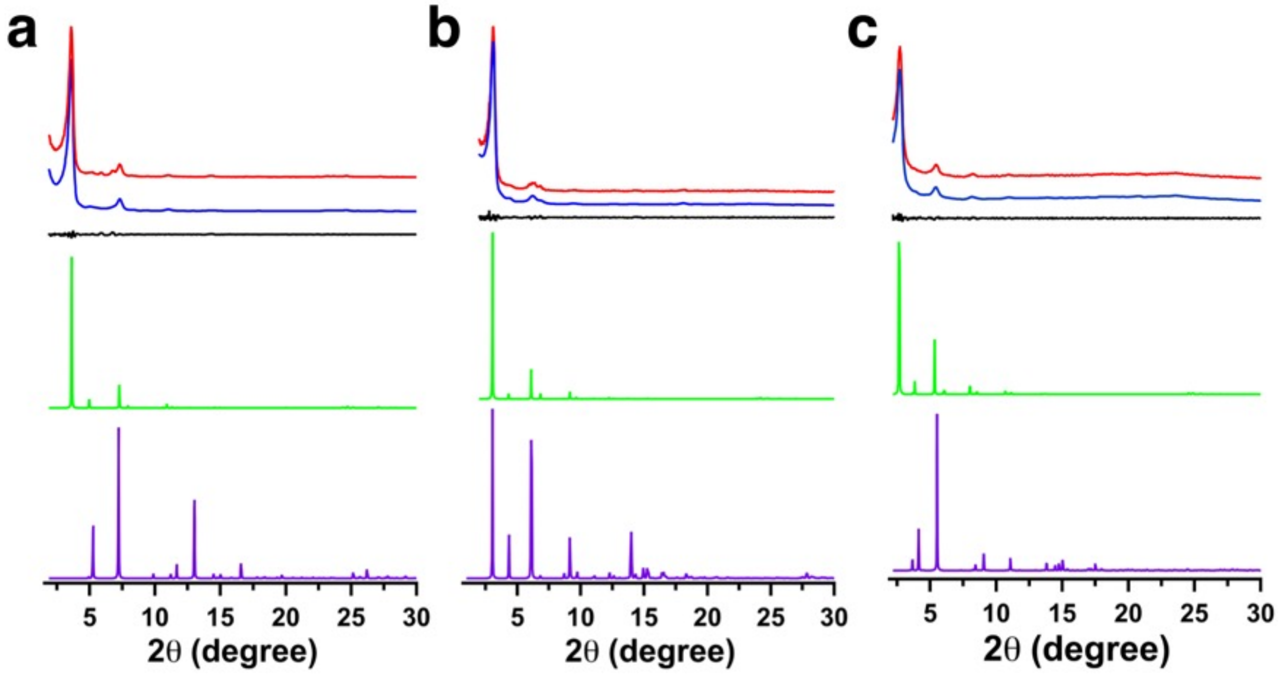

Supplementary Figure 10 | a-c, PXRD profiles of (**a**) sp^{2}c-COF, (**b**) sp^{2}c-COF-2 and (**c**) sp^{2}c-COF-3. Experimentally observed (red curve), Pawley refined (blue curve), their difference (black curve), simulated AA stacking mode (green curve) and simulated AB stacking mode (purple curve).

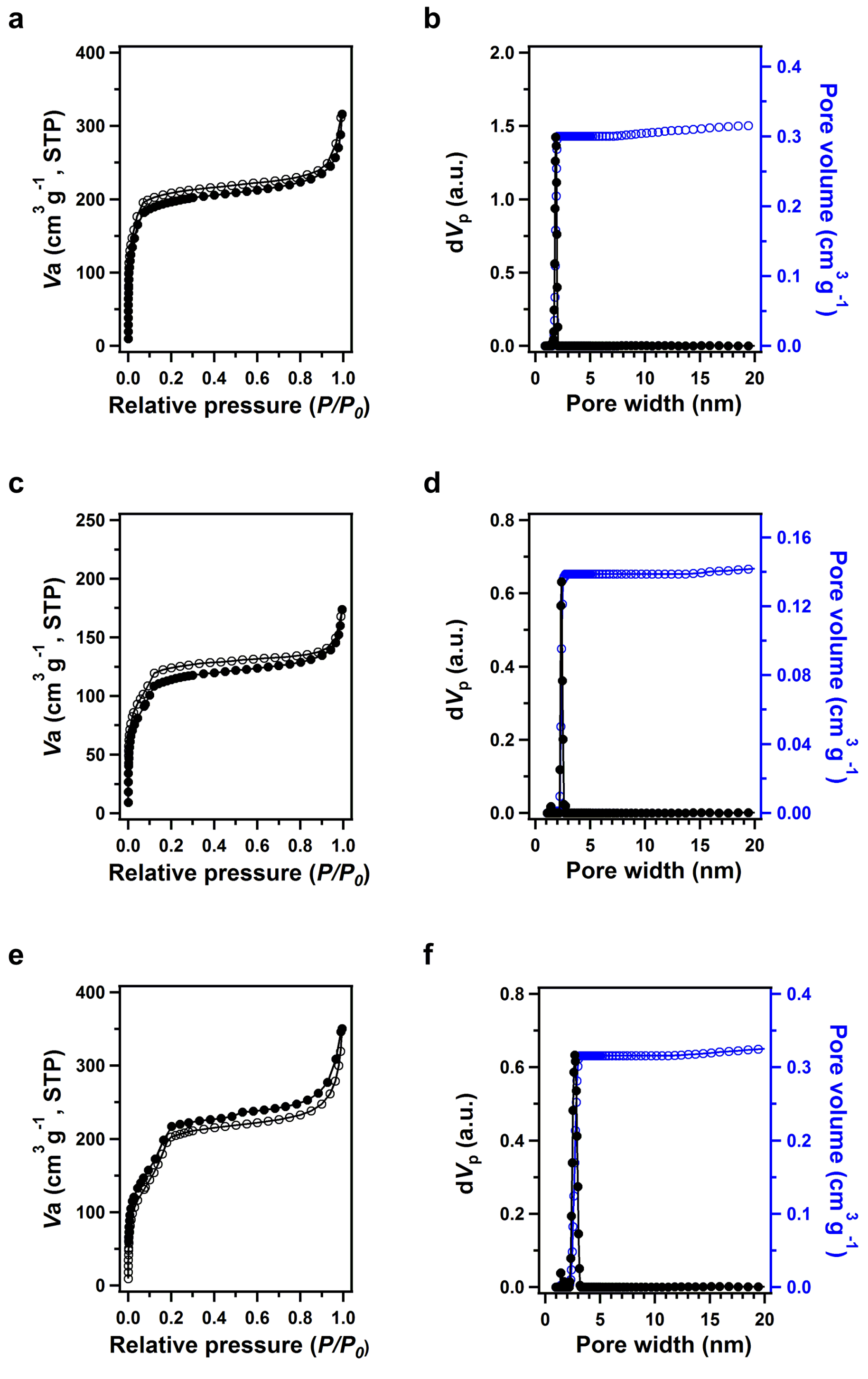

Supplementary Figure 11 | Porosity. The BET surface areas of (a) sp^2c-COF, (c) sp^2c-COF-2 and (e) sp^2c-COF-3. Pore size distribution profiles of (b) sp^2c-COF, (d) sp^2c-COF-2 and (f) sp^2c-COF-3.

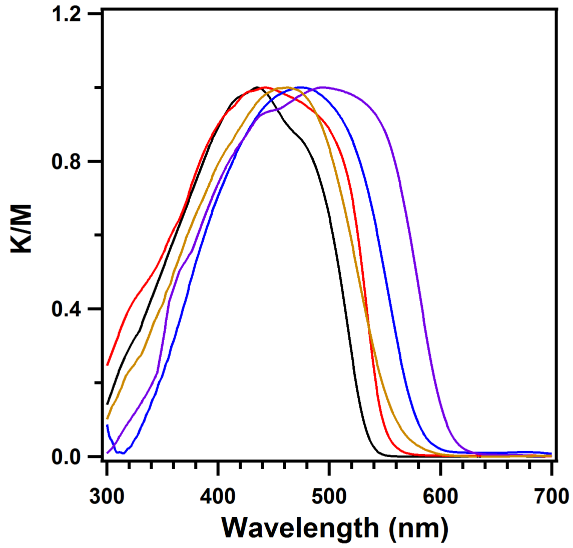

Supplementary Figure 12 | Solid-state electronic absorption spectra (K/M spectra) of the solid samples. Electronic absorption spectra of TFPPy monomer (black curve), model compound (red curve), sp^{2}c-COF (purple curve), sp^{2}c-COF-2 (blue curve) and sp^{2}c-COF-3 (orange curve).

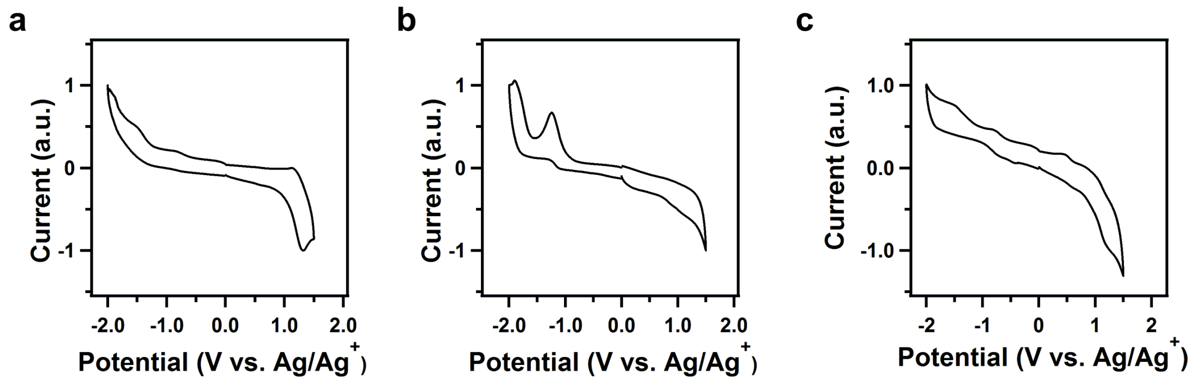

Supplementary Figure 13 | CV curves of sp^{2}c-COFs dispersed solution dropped on carbon glassy electrode. CV curves of (a) sp^{2}c-COF, (b) sp^{2}c-COF-2 and (c) sp^{2}c-COF-3.

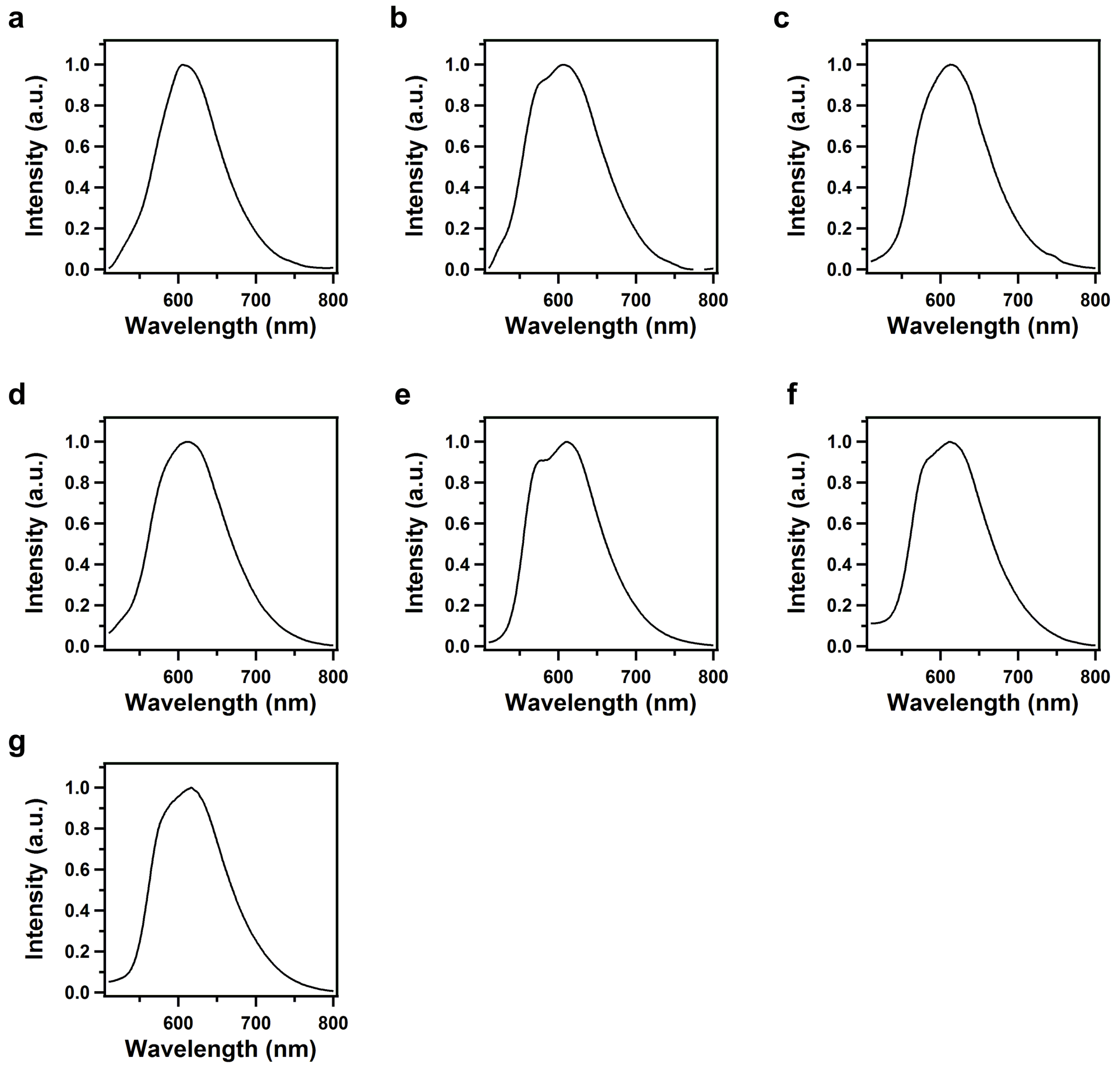

Supplementary Figure 14 | Fluorescence spectroscopy of sp^{2}c-COF dispersed in different solvents. Fluorescence spectra of sp^{2}c-COF dispersed in different solvents of (a) H_2O, (b) CH_3CN, (c) hexane, (d) MeOH, (e) toluene, (f) CH_2Cl_2 and (g) THF.

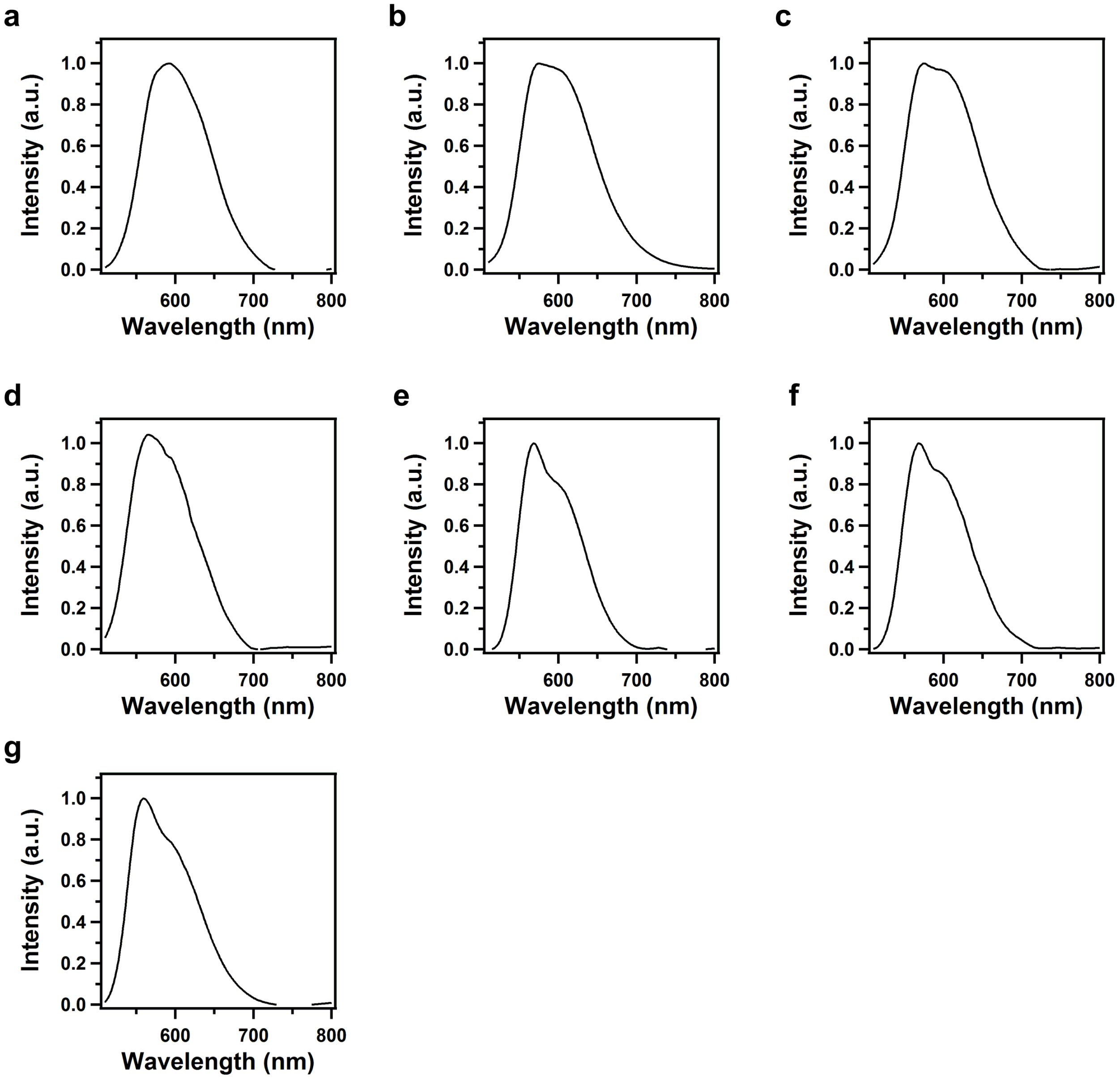

Supplementary Figure 15 | Fluorescence spectroscopy of sp^{2}c-COF-2 dispersed in different solvents. Fluorescence spectra of sp^{2}c-COF-2 dispersed in different solvents of (a) H_2O, (b) CH_3CN, (c) hexane, (d) MeOH, (e) toluene, (f) CH_2Cl_2 and (g) THF.

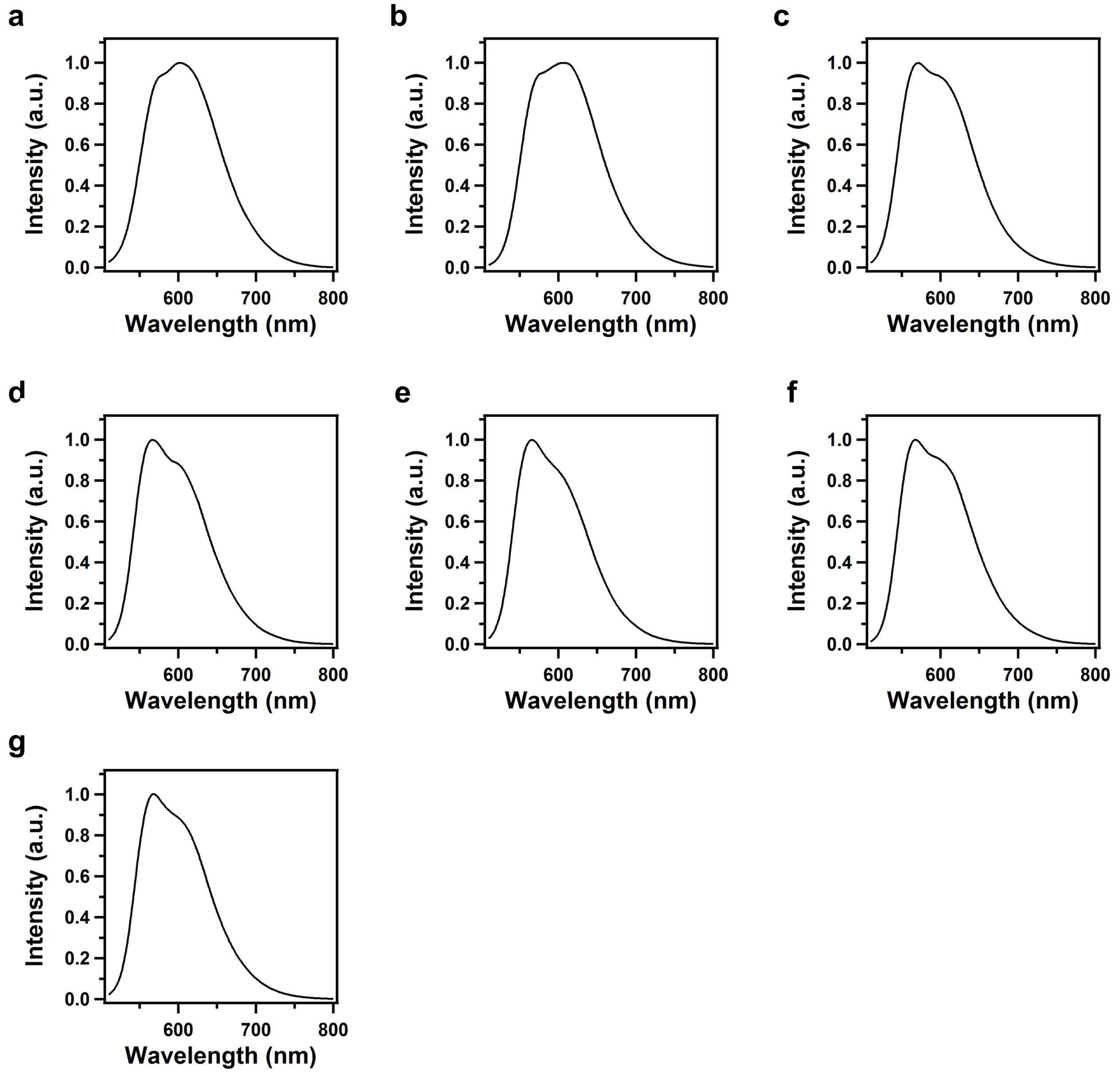

Supplementary Figure 16 | Fluorescence spectroscopy of sp^{2}c-COF-3 dispersed in different solvents. Fluorescence spectra of sp^{2}c-COF-3 dispersed in different solvents of (a) H_2O, (b) CH_3CN, (c) hexane, (d) MeOH, (e) toluene, (f) CH_2Cl_2 and (g) THF.

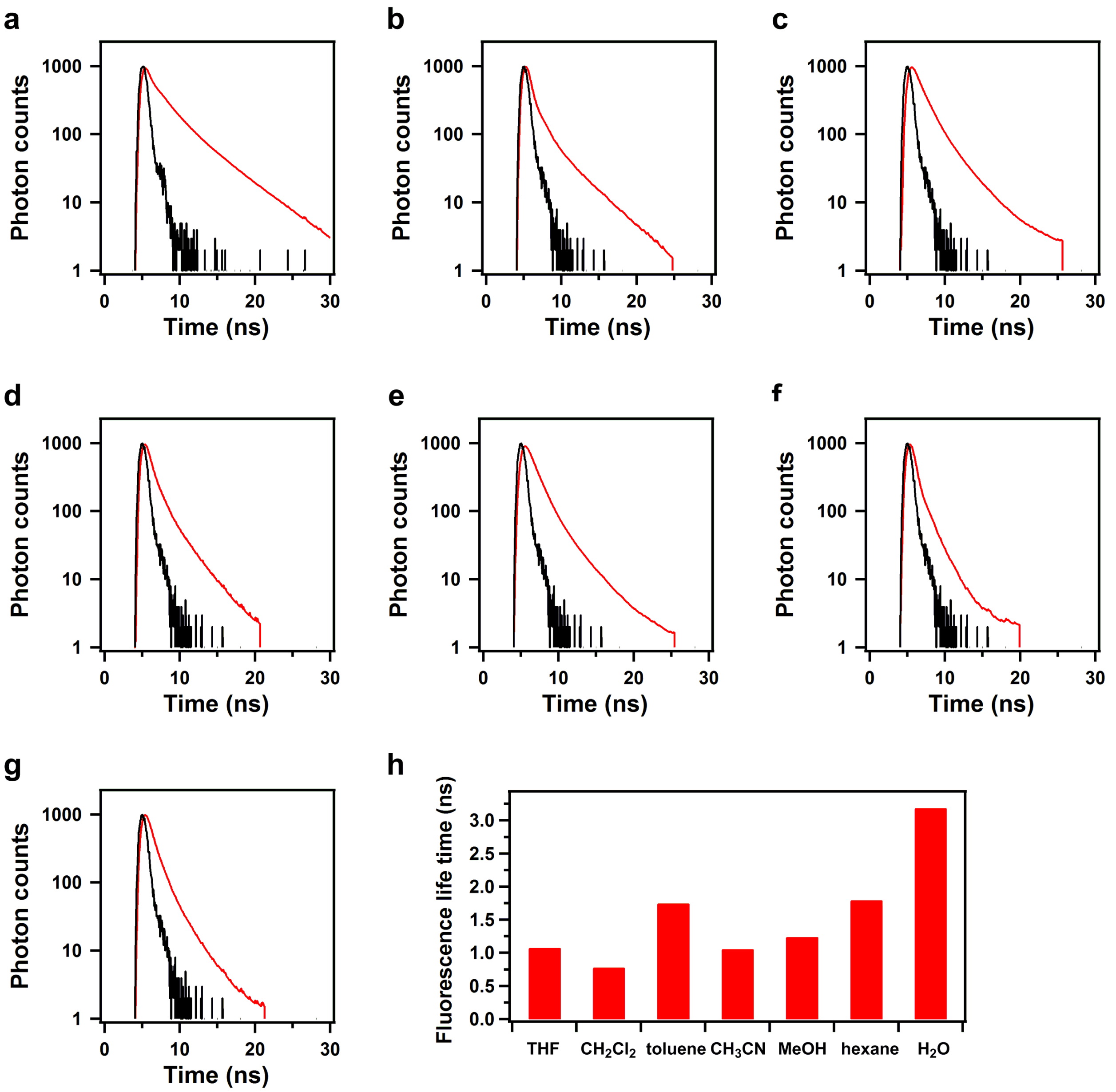

Supplementary Figure 17 | Fluorescence lifetime of sp^{2}c-COF dispersed in different solvents. **a-g,** Fluorescence decay files (red curves) of sp^{2}c-COF dispersed in different solvents of (a) H_2O, (b) CH_3CN, (c) hexane, (d) MeOH, (e) toluene, (f) CH_2Cl_2 and (g) THF. The black curve is the instrumental response function. **h,** Fluorescence lifetime of sp^{2}c-COF in different solvents.

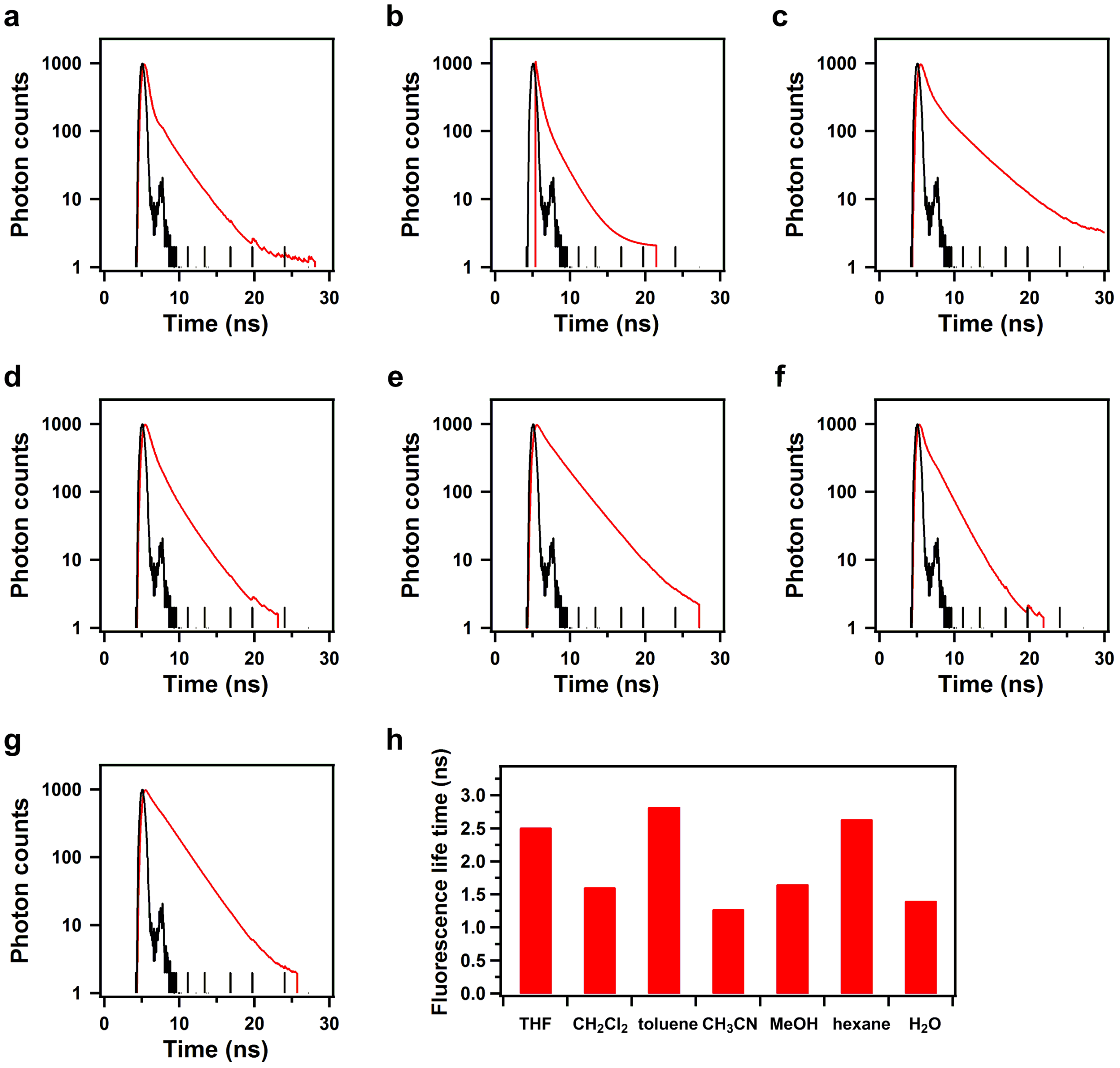

Supplementary Figure 18 | Fluorescence lifetime of sp^{2}c-COF-2 dispersed in different solvents. a-g, Fluorescence decay files (red curves) of sp^{2}c-COF-2 dispersed in different solvents of (a) H_2O, (b) CH_3CN, (c) hexane, (d) MeOH, (e) toluene, (f) CH_2Cl_2 and (g) THF. The black curve is the instrumental response function. **h,** Fluorescence lifetime of sp^{2}c-COF-2 in different solvents.

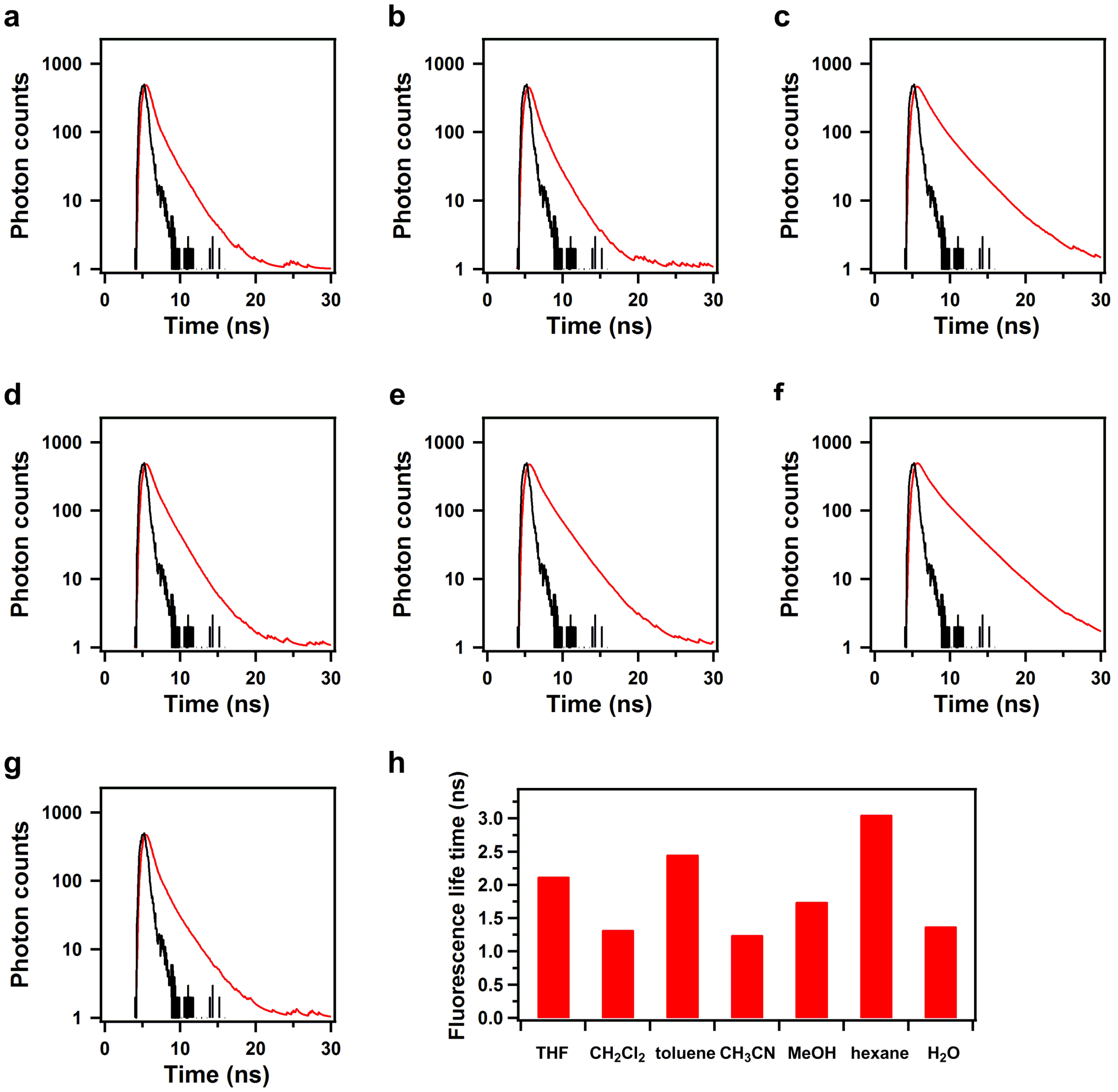

Supplementary Figure 19 | Fluorescence lifetime of sp^{2}**c-COF-3 dispersed in different solvents. a-g,** Fluorescence decay files (red curves) of sp^{2}c-COF dispersed in different solvents of (a) H_2O, (b) CH_3CN, (c) hexane, (d) MeOH, (e) toluene, (f) CH_2Cl_2 and (g) THF. The black curve is the instrumental response function. **h,** Fluorescence lifetime of sp^{2}c-COF in different solvents.

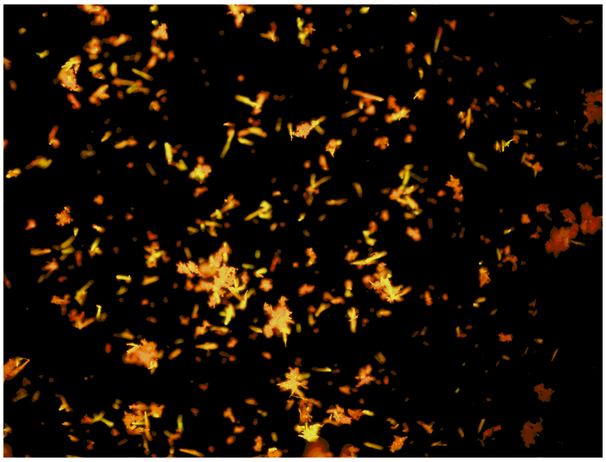

Supplementary Figure 20 | Fluorescence microscopic image of sp^{2}c-COF.

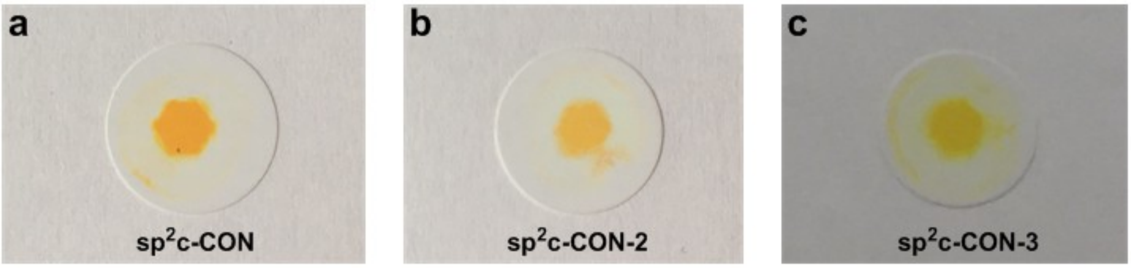

Supplementary Figure 21 | Photo images of sp^2c-CONs thin films. a, A sp^2c-CON film exfoliated from sp^2c-COF. **b,** A sp^2c-CON-2 film exfoliated from sp^2c-COF-2. **c,** A sp^2c-CON-3 film exfoliated from sp^2c-COF-3.

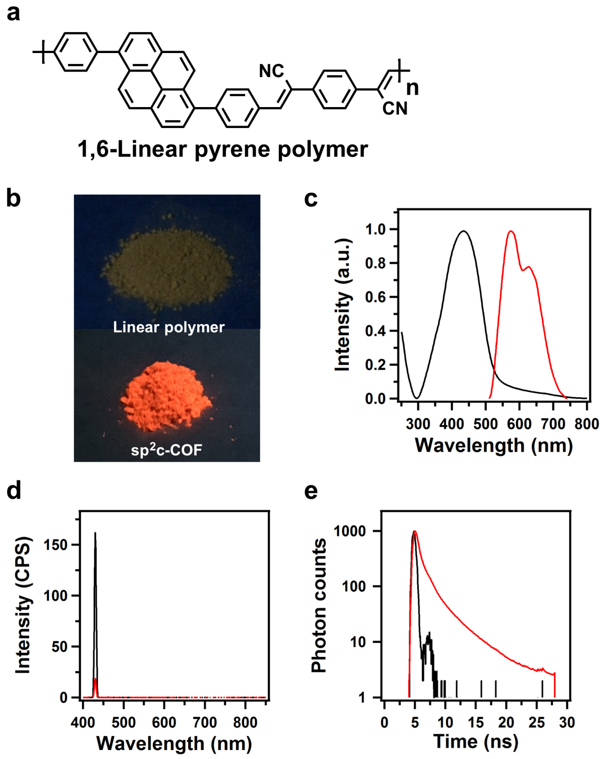

Supplementary Figure 22 | 1,6-Linear pyrene polymer. a, Structure of 1,6-linear pyrene polymer. b, Images of 1,6-linear pyrene polymer and sp^{2}c-COF under UV lamp. **c,** Solid-sate electronic absorption (K/M) and fluorescence spectra. **d,** Spectrum of solid-state absolute quantum yield measured using integral sphere. **e,** Fluorescence decay profile (red curve). Black curve is the instrumental response function.

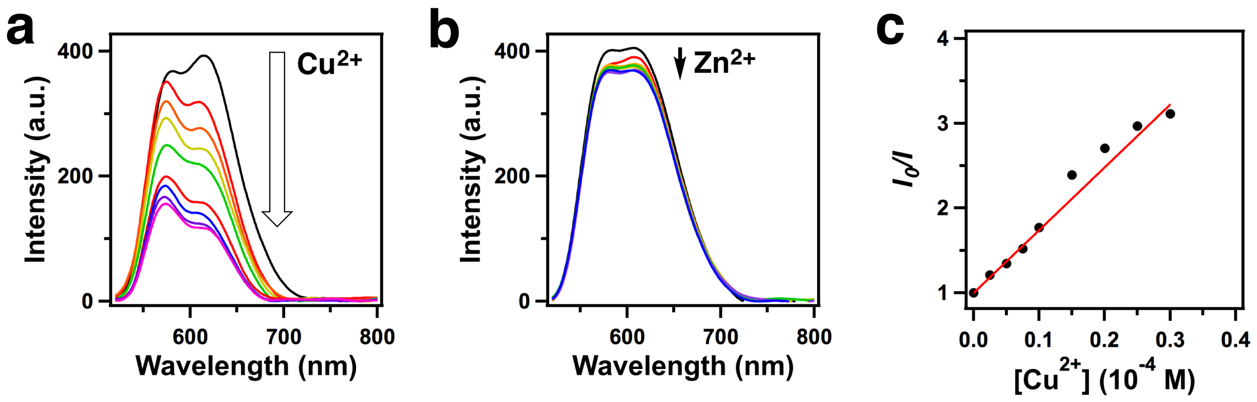

Supplementary Figure 23 | Fluorescence sensor for metal ions. a, b, Fluorescence spectral change of sp^{2}c-COF upon addition of (a) Cu^{2+} and (b) Zn^{2+} ions. **c,** Stern-Volmer plot of the fluorescence quenching by Cu^{2+} ion.

Supplementary Methods

Fourier transform infrared (FT IR) spectra were recorded on a JASCO model FT-IR-6100 infrared spectrometer. Powder X-ray diffraction (PXRD) data were recorded on a Rigaku model RINT Ultima III diffractometer by depositing powder on glass substrate. Nitrogen sorption isotherms were measured at 77 K with a Micromeritics Instrument Corporation model 3Flex surface characterization analyzer. The Brunauer-Emmett-Teller (BET) method was utilized to calculate the specific surface areas. By using the non-local density functional theory (NLDFT) model, the pore volume was derived from the sorption curve. TGA measurements were performed on a Mettler-Toledo model TGA/SDTA851e under N_2, by heating to 800 °C at a rate of 10 °C min^{-1}. Field-emission scanning electron microscopy (FE-SEM) was performed on a JEOL model JSM-6700 and/or Hitachi high technologies (SU-6600) operating at an accelerating voltage of 5.0 kV. The sample was prepared by drop-casting anhydrous acetone suspension onto mica substrate and then coated with gold. High-resolution transmission electron microscopy (HR-TEM) images were obtained on a JEOL model JEM-3200 microscopy. The sample was prepared by drop-casting a supersonicated tetrahydrofuran suspension of the COFs onto a copper grid. Elemental analysis was performed on a Yanako CHN CORDER MT-6 elemental analyzer.

The crystalline structures of COF were determined using the density-functional tight-binding (DFTB+) method including Lennard-Jones (LJ) dispersion. The calculations were carried out with the DFTB+ program package version 1.2[1]. DFTB is an approximate density functional theory method based on the tight binding approach and utilizes an optimized minimal LCAO Slater-type all-valence basis set in combination with a two-center approximation for Hamiltonian matrix elements. The Coulombic interaction between partial atomic charges was determined using the self-consistent charge (SCC) formalism. Lennard-Jones type dispersion was employed in all calculations to describe van der Waals (vdW) and π-stacking interactions. The lattice dimensions were optimized simultaneously with the geometry. Standard DFTB parameters for X–Y element pair (X, Y = C, O, H and N) interactions were employed from the mio-0-1 set[1]. The accessible surface areas were calculated from the Monte Carlo integration technique using a nitrogen-size probe molecule (diameter = 3.68 Å) roll over the framework surface with a grid interval of 0.25 Å.

The XRD pattern simulation was performed in a software package for crystal determination from XRD pattern, implemented in MS modeling version 4.4 (Accelrys Inc.). We performed Pawley refinement to optimize the lattice parameters iteratively until the R_P and R_{WP} values converge. The

pseudo-Voigt profile function was used for whole profile fitting and Berrar-Baldinozzi function was used for asymmetry correction during the refinement processes.

Dehydrated *N,N*-dimethylformide (DMF), dehydrated tetrahydrofuran (THF), toluene, mesitylene, 1,4-dioxane, *o*-dichlorobenzene (*o*-DCB), 1-butanol, ethanol, and concentrated hydrochloric acid were purchased from Wako Chemicals.

Exfoliation procedure. The sp^2c-COFs sample (25 mL vial, 0.1 mg/mL) was sonicated using bath sonic (Branson Sonifier 250) for 2 min. The resulting solution was sonicated using probe sonication (Sonic Power = 250 W, Duty Cycle = 10, Output = 5) in a pulse mode in an ice bath for 30 min. After probe sonication, the dispersion was kept to stand for 20 min and was centrifuged at 1000 rpm for 1 h. The supernatant was collected with a pipette. To fabricate thin films, the supernatant (5 mL) was filtered through a 20-nm-pore AAO filter disk.

Fluorescence sensing. A Cu^{2+} stock solution (3×10^{-4} mol L^{-1}) was prepared by dissolving $CuCl_2$ (4.03 mg) in acetonitrile (100 mL). A stock solution of sp^2c-COF was prepared by dispersing COF (0.28 mg) in a mixture of dichloromethane and acetonitrile (v/v = 1/3, 40 mL). A well dispersed solution (2.7 mL) of sp^2c-COF was placed in a quartz cell and fluorescence spectra were recorded immediately after the addition of the Cu^{2+} solution of 25, 50, 75, 100, 150, 200, 250 and 300 μL, upon excitation at 498 nm. A certain amount of solvent mixture of dichloromethane and acetonitrile (v/v = 1/3) was added to the quartz cell so that the total solution volume was kept at a constant (3 mL). Each quenching point was repeated at least for three times to get the reliable value. The sp^2c-COF suspension exhibited a rapid response to the Cu^{2+} ion and decreased its luminescence by keeping the shape of the emission spectrum. Stern-Volmer plot revealed a nearly linear curve, whereas the fluorescence quenching rate constant k_q (= k_{SV}/τ) was evaluated to be as high as 4.1×10^{14} M^{-1} s^{-1} (τ is the fluorescence lifetime). The Zn^{2+} stock solution (3×10^{-4} mol/L) was prepared by dissolving $Zn(OAc)_2$ (5.50 mg) in acetonitrile (100 mL), whereas other operations were similar to that of the Cu^{2+} detection.

Supplementary References

1. Aradi, B., Hourahine, B. & Frauenheim, T. DFTB+, a sparse matrix-based implementation of the DFTB method. *J. Phys. Chem. A* **111**, 5678-5684 (2007).
